# Impaired AGO2/miR-185-3p/NRP1 axis promotes colorectal cancer metastasis

**DOI:** 10.1038/s41419-021-03672-1

**Published:** 2021-04-12

**Authors:** Xisheng Liu, Xiaole Meng, Xiao Peng, Qianlan Yao, Fangming Zhu, Zhongyi Ding, Hongze Sun, Xueni Liu, Dan Li, Ying Lu, Huamei Tang, Bin Li, Zhihai Peng

**Affiliations:** 1 Department of General Surgery, Shanghai General Hospital, Shanghai Jiao Tong University School of Medicine, Shanghai, China; 2 Organ Transplantation Institute of Xiamen University, Fujian Provincial Key Laboratory of Organ and Tissue Regeneration, School of Medicine, Xiamen University, Xiamen, China; 3 Department of Biology, Temple University, Philadelphia, PA USA; 4 Department of Pathology, Fudan University Shanghai Cancer Center, Shanghai, China; 5 Department of Oncology, Shanghai Medical College, Fudan University, Shanghai, China; 6 Department of Microbiology, School of Medicine, University of Alabama at Birmingham, Birmingham, AL USA; 7 Laboratory Animal Center, Institute Pasteur of Shanghai, University of Chinese Academy of Sciences, Shanghai, China; 8 Shanghai Institute of Immunology and Department of Immunology and Microbiology, Shanghai Jiao Tong University School of Medicine, Shanghai, China; 9 Department of Biochemistry and Molecular Cell Biology, Shanghai Key Laboratory for Tumor Microenvironment and Inflammation, Shanghai Jiao Tong University School of Medicine, Shanghai, China; 10 Department of Pathology, Xiang’an Hospital of Xiamen University, Xiamen, China; 11 Hepatobiliary and Pancreatic & Organ Transplantation Surgery Department, Xiang’an Hospital of Xiamen University, Xiamen, China

**Keywords:** Colorectal cancer, Proteomics

## Abstract

Increasing evidence suggests that global downregulation of miRNA expression is a hallmark of human cancer, potentially due to defects in the miRNA processing machinery. In this study, we found that the protein expression of Argonaute 2 (AGO2), a key regulator of miRNA processing, was downregulated in colorectal cancer (CRC) tissues, which was also consistent with the findings of the Clinical Proteomic Tumor Analysis Consortium (CPTAC). Furthermore, the correlation between the levels of AGO2 and epithelial-mesenchymal transition (EMT) markers (E-cadherin and vimentin) indicated that reduced levels of AGO2 promoted EMT in CRC. Low expression of AGO2 was an indicator of a poor prognosis among CRC patients. Knockdown of AGO2 in CRC cells promoted migration, invasion and metastasis formation in vitro and in vivo but had no influence on proliferation. To provide detailed insight into the regulatory roles of AGO2, we performed integrated transcriptomic, quantitative proteomic and microRNA sequencing (miRNA-seq) analyses of AGO2 knockdown cells and the corresponding wild-type cells and identified *neuropilin 1* (*NRP1*) as a new substrate of AGO2 via miR-185-3p. Our data provided evidence that knockdown of AGO2 resulted in a reduction of miR-185-3p expression, leading to the upregulation of the expression of NRP1, which is a direct target of miR-185-3p, and elevated CRC cell metastatic capacity. Inhibition of NRP1 or treatment with a miR-185-3p mimic successfully rescued the phenotypes of impaired AGO2, which suggested that therapeutically targeting the AGO2/miR-185-3p/NRP1 axis may be a potential treatment approach for CRC.

## Introduction

Colorectal cancer (CRC) is the world’s fourth most deadly cancer, with almost 900,000 deaths occurring per year^1^. The 5-year survival rate for CRC is over 90% for stage I disease, but it is below 10% when CRC develops into advanced stage IV disease with metastasis^2^. When CRC progresses to advanced stages, especially with fatal distant metastases, targeted therapies are an essential component of the comprehensive treatment regimen^3^. Therefore, it is urgent to elucidate the pathogenic mechanisms by which cancer cells migrate, invade, and cope with the tumor microenvironment, and to identify biomarkers to predict the occurrence of metastasis and that are potential targets of treatments to halt these processes.

Mounting evidence suggests that microRNAs (miRNAs) play a critical role in tumorigenesis. Most types of tumors show a global reduction in miRNA expression, which is closely associated with cancer development^4^. These findings indicate that although some specific miRNAs serve as oncogenes, miRNAs generally play an essential role in tumor suppression. The global reduction of miRNAs in cancers does not coincide with reductions in their primary transcripts^5^, which suggests that altered regulation of the miRNA-processing machine may be responsible for the reduction of mature miRNA. Dysregulation of miRNA-processing machinery components plays a crucial role in cancer^6^ and serves as a prognostic marker^7^.

Argonaute 2 (AGO2) is a key regulator of miRNA processing and is implicated in tumourigenesis^8^. As one of four mammalian AGO family proteins, AGO2 is ubiquitously expressed and functions as a unique protein of the RNA-induced silencing complex (RISC) with endoribonuclease (“slicer”) activity^9^. AGO2 can generate mature miRNA in a slicer-dependent^10^ or slicer-independent manner^11^ and is able to protect miRNAs from degradation^12–14^. The impairment of AGO2 results in globally decreased expression of miRNAs^15^.

Many studies have focused on the role of AGO2 in tumorigenesis and tumor progression. Upregulation of AGO2 has been identified in multiple tumor types, such as breast cancer^16^, colon cancer^17^, myeloma^18^, hepatocellular carcinoma^19^, gastric carcinoma^20^, and non-melanoma skin cancer^21^. Paradoxically, however, some other studies also reported that AGO2 suppresses tumor growth and/or metastasis in lung adenocarcinoma^22^, melanoma^23^, breast carcinoma^24,25^, and non-small cell lung carcinomas^26^. Genetic variants in the AGO2 gene lead to the loss of AGO2 expression, including frameshift mutations in the AGO2 gene with high microsatellite instability (MSI-H) in gastric cancer and CRC^27^, single nucleotide polymorphisms of AGO2 in breast cancer^28^, and AGO2 mutations in glioma^29^. In this research, we studied the function of AGO2 in CRC tumor specimens and CRC cells and explored the possible molecular mechanism of AGO2 in CRC.

## Results

### Reduced expression of AGO2 protein is an indicator of a poor prognosis in CRC patients

**Fig. 1:**
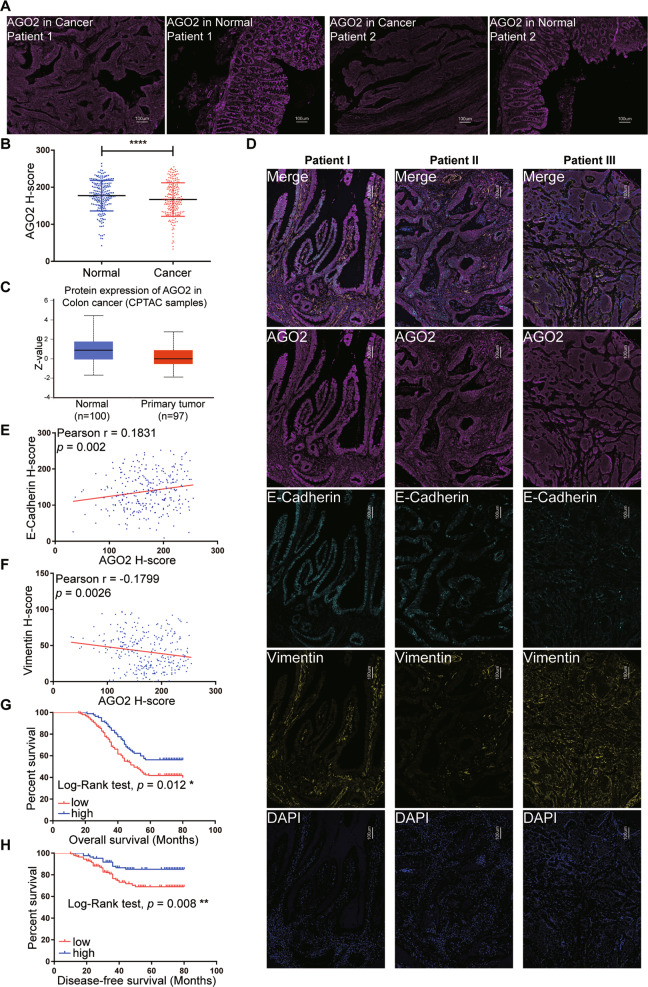
Reduced expression of AGO2 indicates a poor prognosis in CRC patients. **A** Representative multispectral IHC staining images of AGO2 protein. The scale bars are 100 μm. **B** Comparison of the levels of AGO2 in CRC cancer tissues and normal tissues. **C** The protein level of AGO2 analysed in primary colon tumor tissues and normal tissues from the CPTAC data. *Z*-values represent standard deviations from the median across samples for the given cancer type. **D** Multispectral IHC staining images for AGO2, E-cadherin, vimentin, and DAPI in cancer tissues. The scale bars are 100 μm. **E**, **F** Pearson correlation analysis between the H-scores of AGO2 and E-cadherin (**E**) and AGO2 and vimentin (**F**). **G**, **H** Kaplan–Meier plots for the OS (**G**) and DFS (**H**) of 284 CRC patients who underwent curative resections. Data are expressed as the mean ± SD. *p < 0.05, **p < 0.01, and ****p < 0.0001.

To determine the potential role of AGO2 in CRC, we measured the expression of AGO2 in 213 paired cancer tissues and corresponding normal tissues by multiplex fluorescent IHC and a scoring system based on InForm software^30^ (Fig. S1A, B). We found that AGO2 was significantly expressed at lower levels in cancer tissues (Fig. 1A, B). And the expression of AGO2 was significantly higher in cancer tissues than in stromal tissues (Fig. S1C). Consistently, we found that the AGO2 protein was downregulated in primary colon tumor tissues in the CPTAC data (Fig. 1C)^31^. It has been widely accepted that epithelial–mesenchymal transition (EMT) is associated with the loss of E-cadherin and the upregulation of vimentin^32^. Then, we examined the expression levels of AGO2, E-cadherin, and vimentin in 284 colorectal cancer tissue samples (Fig. 1D). We found a significantly positive correlation between E-cadherin and AGO2 protein levels (*r* = 0.1831, *p* = 0.0020) (Fig. 1E), but a negative correlation between vimentin and AGO2 protein levels in CRC (*r* = −0.1799, *p* = 0.0026) (Fig. 1F). Furthermore, we found correlations between AGO2 expression and the clinicopathological features of CRC, as shown in Table 1. Based on AGO2 immunoreactivity, 198 samples (69.7%) exhibited low AGO2 expression (*H*-score < 200), and 86 samples (30.3%) exhibited high AGO2 expression (*H*-score ≥ 200). CRC patients with higher cancer stage (*p* = 0.009), lymph node metastasis (*p* = 0.022), and distant metastasis (*p* < 0.001) exhibited lower AGO2 expression. Kaplan–Meier analyses revealed that patients with low expression of AGO2 had a shorter overall survival (OS) and disease-free survival (DFS) than patients with high expression of AGO2 (Fig. 1G, H). Univariate and multivariate Cox proportional hazard regression analyses showed that low expression of AGO2 was an independent predictor of a shorter DFS (hazard ratio, 2.200 [1.176–4.116]; *p* = 0.014) and OS (hazard ratio, 1.559 [1.076–2.258]; *p* = 0.019) (Tables S2 and S3). Taken together, low expression of AGO2 is an independent predictor of a poor outcome, and the downregulation of AGO2 might promote EMT in CRC.

**Table 1:** Relationship between clinical features and AGO2 protein expression in 284 colorectal cancer patients.

Parameters	*n* (%)	AGO2 expression		
		Low	High	*p*
*Gender*				0.627
Male	135 (47.5)	96	39	
Female	149 (52.5)	102	47	
*Age, years*				0.427
<65	109 (38.4)	73	36	
≥65	175 (61.6)	125	50	
*Tumor site*				0.885
Right	54 (19.0)	35	19	
Transverse	30 (10.6)	20	10	
Left	33 (11.6)	23	10	
Sigmoid	83 (29.2)	59	24	
Rectal	84 (29.6)	61	23	
*Tumor size*				0.774
<5 cm	112 (39.4)	77	35	
≥5 cm	172 (60.6)	121	51	
*Tumor grade*				0.361
Well/Moderate	218 (76.8)	149	69	
Poor	66 (23.2)	49	17	
*pT stage*				0.250
T1-2	73 (25.7)	47	26	
T3-4	2111 (74.3)	151	60	
*pN stage*				0.022
N0	149 (52.5)	95	54	
N1-2	135 (47.5)	103	32	
*pM stage*				<0.001
M0	242 (85.2)	157	85	
M1	42 (14.8)	41	1	
*AJCC stage*				0.009
I–II	145 (51.1)	91	54	
III–IV	139 (48.9)	107	32	

*P* < 0.05 indicates a significant association among the variables.

### AGO2 has no effect on the growth of CRC cells

**Fig. 2:**
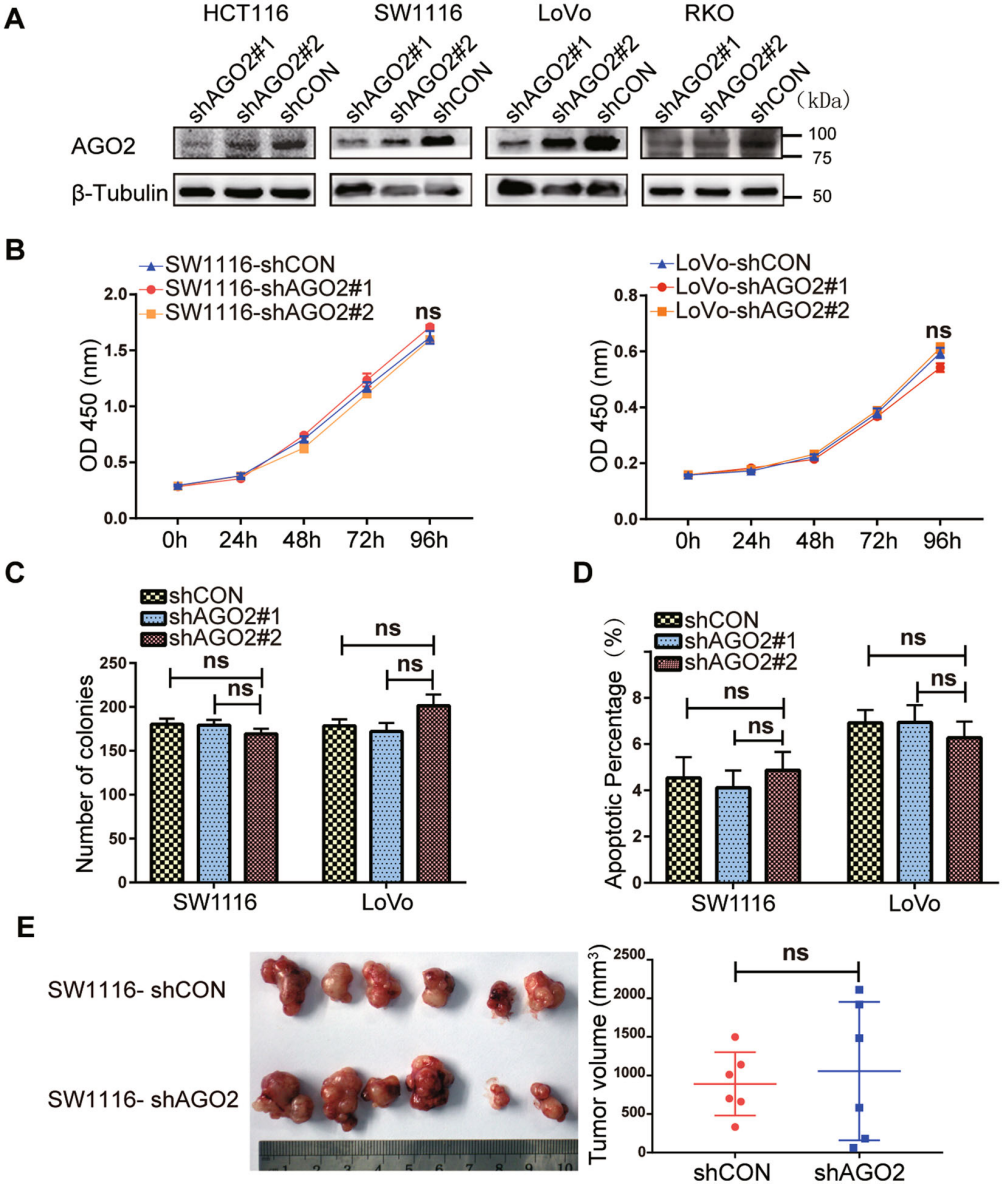
AGO2 knockdown has no influence on the growth of CRC cells. **A** Representative immunoblots for AGO2 expression in CRC cell lines stably transfected with shAGO2 or shCON. **B** CCK-8 assay in SW1116 (left) and LoVo (right) cells. **C** Statistical analysis of colony formation assays. **D** Statistical analysis of early and late apoptotic cells. **E** Representative photographs (left) and statistical analysis (right) of tumor xenografts in mice (n = 6 per group). Data are expressed as the mean ± SD, ns no significance, *p* > 0.05.

Then, to assess whether AGO2 is involved in malignant progression, we measured the protein levels of AGO2 in different CRC cell lines (Fig. S2A). Among the four AGO proteins, only AGO2 has slicer activity that can be used for shRNA-mediated knockdown^33^, and it is intrinsically difficult to achieve effective knockdown when the target is AGO2 itself. Fortunately, the efficiency of AGO2 knockdown in CRC cells was successfully confirmed (Fig. 2A). However, the results of CCK-8 assays revealed that the knockdown of AGO2 had no influence on cell viability (Fig. 2B). Colony formation assays also showed that knockdown of AGO2 had no effect on cell proliferation (Fig. 2C and Fig. S2B). Flow cytometry assays demonstrated that the knockdown of AGO2 had no effect on the apoptosis of CRC cells (Fig. 2D and Fig. S2C). We also established xenograft tumors in mice and found that the tumor volume of the AGO2-knockdown group was not significantly different from that of the control group (Fig. 2E). Furthermore, we constructed CRC cells stably overexpressing AGO2 (Fig. S2D). Parallel analyses revealed that there were no significant effects on the growth of CRC cells overexpressing AGO2 (Fig. S2E–G). These findings indicate that AGO2 does not affect the growth of CRC cells either in vitro or in vivo.

### AGO2 knockdown promotes the migration, invasion and metastasis of CRC cells

**Fig. 3:**
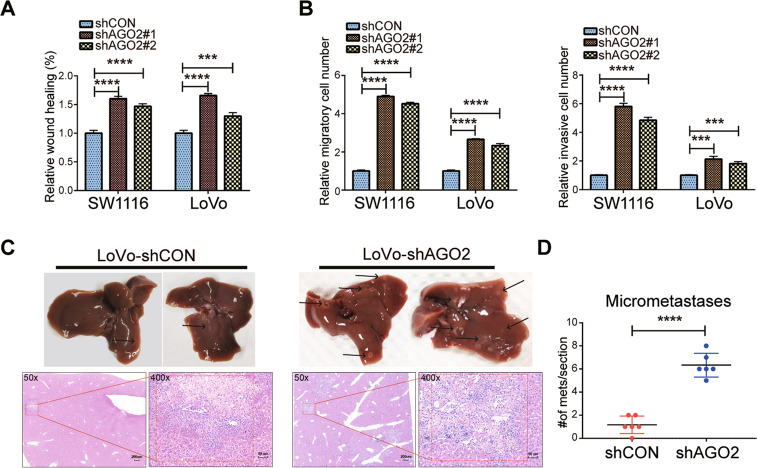
AGO2 knockdown promotes the migration, invasion, and metastasis of CRC cells. **A**, **B** Statistical analyses of cell migration and invasion were determined by wound healing (**A**) and Transwell assays (**B**) upon AGO2 knockdown in CRC cells. **C** Metastatic foci (upper) and H&E-staining images of metastatic nodules (bottom) from mice are shown; scale bars are 50 and 200 μm. **D** The number of liver metastatic nodules was analysed (*n* = 6 per group). Data are shown as the mean ± SD. ***p < 0.001, and ****p < 0.0001.

The above multiplex fluorescent IHC data suggested that the downregulation of AGO2 might promote EMT in CRC. Consistently, we found that AGO2 knockdown led to increased migration and invasion by CRC cells in wound healing and Transwell assays (Fig. 3A, B and Fig. S3A, B). Furthermore, we constructed a nude mouse metastatic model. LoVo cells stably expressing shCON or shAGO2#1 were injected into the spleens of nude mice. The number and volume of liver metastatic nodules were higher in the AGO2-knockdown group than in the control group (Fig. 3C). Microscopic examination of liver sections revealed many more metastatic subclones in the AGO2-knockdown group (Fig. 3D). Consistently, overexpression of AGO2 inhibited cell migration and invasion (Fig. S3C–F). Taken together, AGO2 functions as an inhibitor of the migration, invasion, and metastasis of CRC cells.

### AGO2 regulates NRP1 expression at both the mRNA and protein levels

**Fig. 4:**
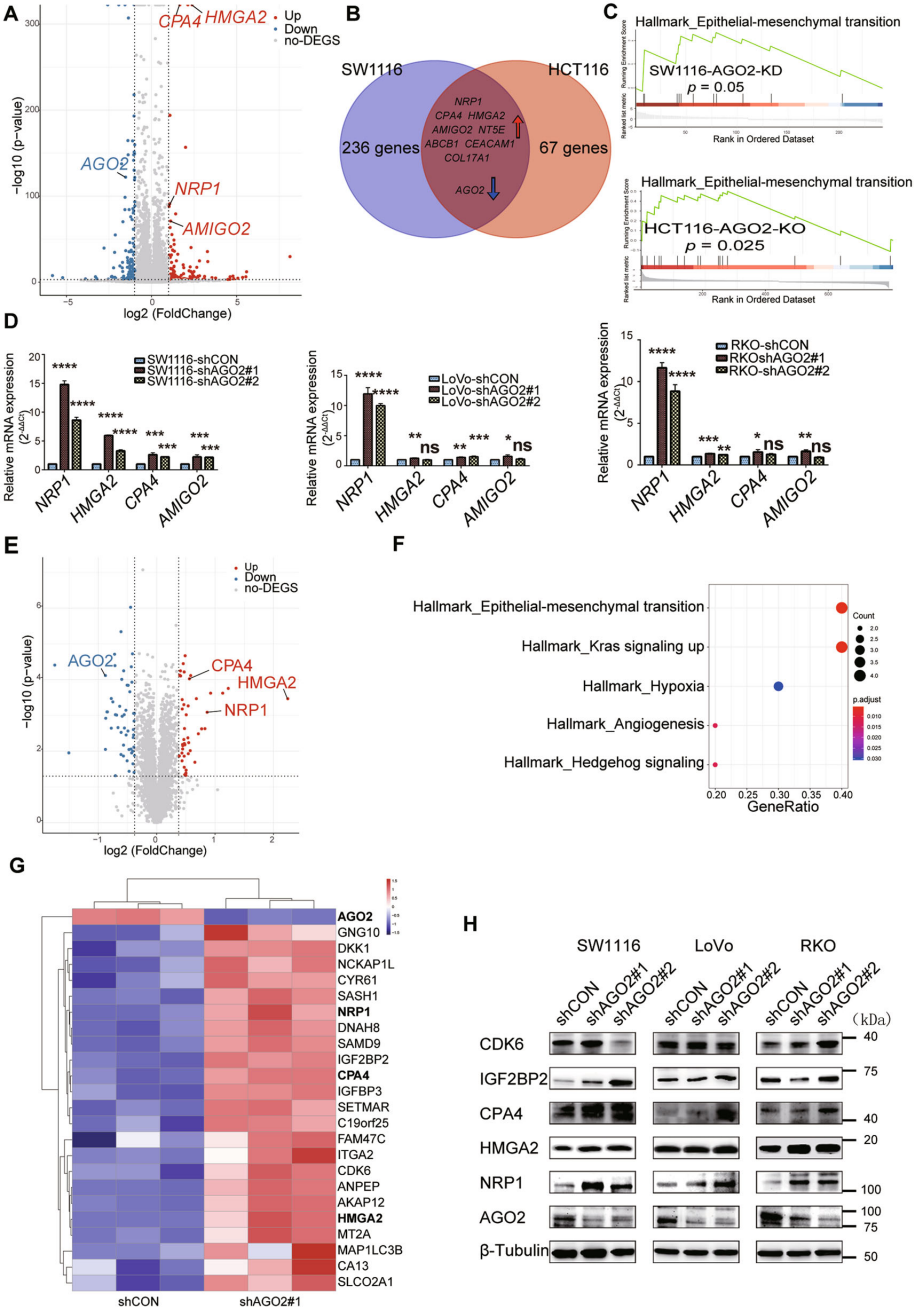
AGO2 regulates the expression of NRP1 at both the mRNA and protein levels in CRC cells. **A** Volcano plot showing transcriptome-wide differences in RNA-seq expression data upon AGO2 knockdown. **B** Venn diagram showing the overlap of RNA-seq expression data upon AGO2 knockdown or AGO2 knockout. **C** GSEA of the transcriptome profiles shows a highly significant enrichment of EMT-induced gene signatures after AGO2 knockdown (upper) or knockout (bottom). **D** The expression of differential genes was determined by qRT-PCR. **E** Volcano plot showing protein differences in quantitative proteomic data upon AGO2 knockdown. **F** GO enrichment analysis of differential expressed proteins. **G** Heatmap of differentially expressed proteins after AGO2 knockdown. **H**. Western blotting analysis of the upregulated oncogenes upon AGO2 knockdown. Data are shown as the mean ± SD. ns (*p* > 0.05), *p < 0.05, **p < 0.01, ***p < 0.001, and ****p < 0.0001.

To investigate the underlying molecular mechanisms of pro-metastatic effect of AGO2 knockdown, we performed RNA-seq on SW1116 cells stably transfected with shAGO2#1 (AGO2-KD) or shCON (AGO2-CON). A total of 245 genes were differentially expressed in AGO2-KD cells (fold change ≥ 2 and *p* ≤ 0.001) compared with their AGO2-CON counterparts, including 135 downregulated genes and 110 upregulated genes (Fig. 4A). Cross-referencing the RNA-seq data to the RNA-seq data of HCT116 AGO2-knockout cells^34^ revealed that NRP1, CPA4, HMGA2, CEACAM1, COL17A1, ABCB1, NT5E, and AMIGO2 were all upregulated, while AGO2 was the only gene downregulated in both datasets (Fig. 4B). Consistent with previous data showing that reduced AGO2 might be involved in EMT (Fig. 1E, F), Gene Set Enrichment Analysis (GSEA) also showed that EMT-related genes were upregulated after AGO2 knockdown or knockout (Fig. 4C). Notably, NRP1 was the most significantly upregulated gene in all three AGO2-knockdown CRC cell lines (Fig. 4D).

Simultaneously, we conducted a quantitative proteomic analysis of SW1116 AGO2-KD and AGO2-CON cells. We found 44 upregulated proteins and 53 downregulated proteins in the AGO2-KD group (Fig. 4E; fold change ≥ 1.3 and *p* ≤ 0.05). Gene Ontology (GO) enrichment analysis revealed pathways involved in EMT and KRAS signaling (Fig. 4F). Integrated analysis of the RNA-seq and proteomic data revealed that NRP1, HMGA2, and CPA4 were upregulated at both the mRNA and protein levels in the AGO2-KD group (Fig. 4G). Western blotting analysis confirmed that the knockdown of AGO2 significantly upregulated the protein level of NRP1 in multiple CRC cell lines, while the expression of the proteins HMGA2, CPA4, CDK6, and IGF2BP2 were changed weakly and inconsistently (Fig. 4H). Furthermore, overexpression of AGO2 significantly inhibited the expression of NRP1 in CRC cells (Fig. S4A). Thus, the AGO2 protein could regulate the expression of NRP1 in CRC cells at both the mRNA and protein levels.

### Downregulation of NRP1 rescues the phenotypes of impaired AGO2

**Fig. 5:**
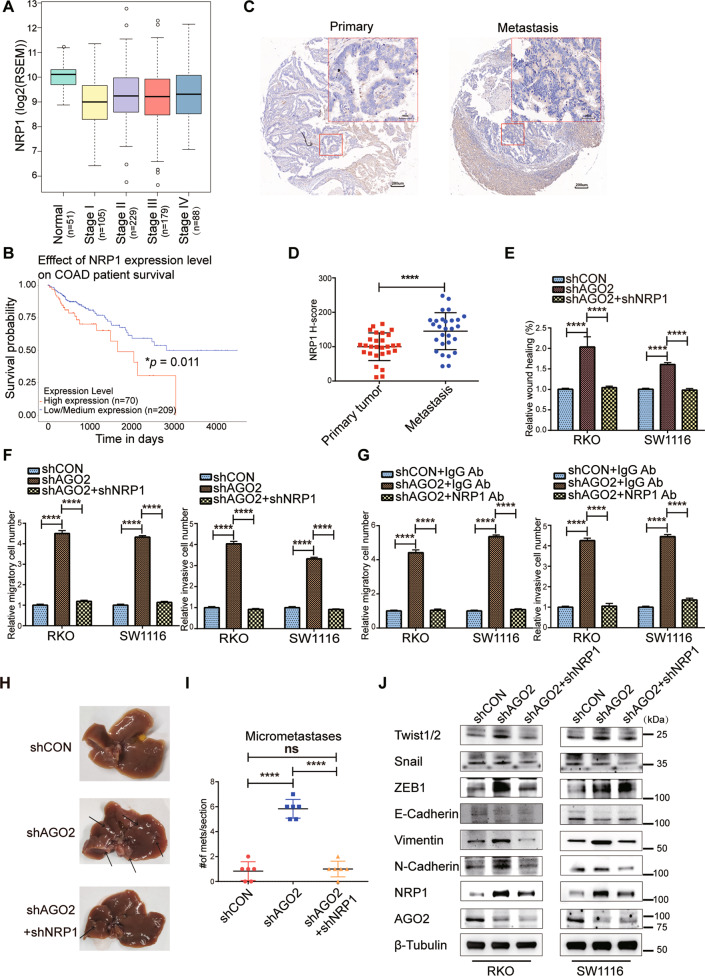
Inhibition of NRP1 rescues the phenotypes of impaired AGO2. **A** The expression of NRP1 in 600 CRC patients from the TCGA database. N: 51, stage I: 105, stage II: 229, stage III: 179, stage IV: 88. **B** Kaplan–Meier plot of the OS of 279 CRC patients from the TCGA database. **C** IHC images of NRP1 in CRC primary tumor tissues (left) and metastatic foci (right); scale bars are 50 and 200 μm. **D** Statistical analysis of H-scores of NRP1 in CRC primary tumor tissues and metastatic foci. **E** Statistical analysis of cell migration upon NRP1 knockdown in AGO2-KD CRC cells. **F**, **G** Statistical analysis of Transwell migration (**F**) and invasion (**G**) upon NRP1 knockdown in AGO2-KD CRC cells. **H** Metastatic foci from the mice are shown. **I** The number of liver metastatic nodules was analysed (*n* = 6 per group). **J** Representative immunoblots of EMT markers. Data are shown as the mean ± SD. *p < 0.05, **p < 0.01, ***p < 0.001, and ****p < 0.0001.

As we found that AGO2 knockdown in CRC cells could promote the expression of NRP1, we presumed that the pro-metastatic effect of impaired AGO2 might be mediated by NRP1. First, we analysed the expression profile of *NRP1* in 600 pairs of CRC tumor tissues and adjacent normal tissues from The Cancer Genome Atlas (TCGA). We found that the mRNA levels of *NRP1* were higher in normal tissues than in tumor tissues, but there was an increasing tendency of NRP1 from stage I to stage IV (Fig. 5A), and patients with high expression of *NRP1* had a poor prognosis (Fig. 5B). We also found that metastatic foci expressed higher levels of NRP1 than primary colorectal tumor tissues (Fig. 5C, D).

As mentioned above, only AGO2 has slicer activity and can be used for shRNA-mediated knockdown^33^. It was difficult to knockdown NRP1 in AGO2-KD cells, but we successfully knocked down NRP1 in SW1116-shAGO2#1 and RKO-shAGO2#1 cell (Fig. S5A). Then, wound healing assay and Transwell assay revealed that the mobility capability of AGO2-KD cells was blocked upon NRP1 knockdown (Fig. 5E, F and Fig. S5B, C). Treatment with a neutralizing anti-NRP1 antibody also significantly decreased the migration and invasion abilities of AGO2-KD cells compared with IgG-treated controls (Fig. 5G and Fig. S5D). We also found that the pro-metastatic effect of impaired AGO2 was blocked when the upregulated NRP1 was inhibited (Fig. 5H, I and Fig. S5E). NRP1 has been reported to interact with numerous ligands and receptors to promote tumor progression and EMT in a number of different cancers^35–37^. Western blotting analysis showed that prototypic EMT markers were significantly altered when NRP1 was knocked down (Fig. 5J). Thus, NRP1 is the functional target of AGO2, and the upregulation of NRP1 is essential for EMT-related pro-metastatic activity in AGO2-knockdown cells.

### AGO2 knockdown reduces the level of miR-185-3p, which direct targets NRP1 in CRC cells

**Fig. 6:**
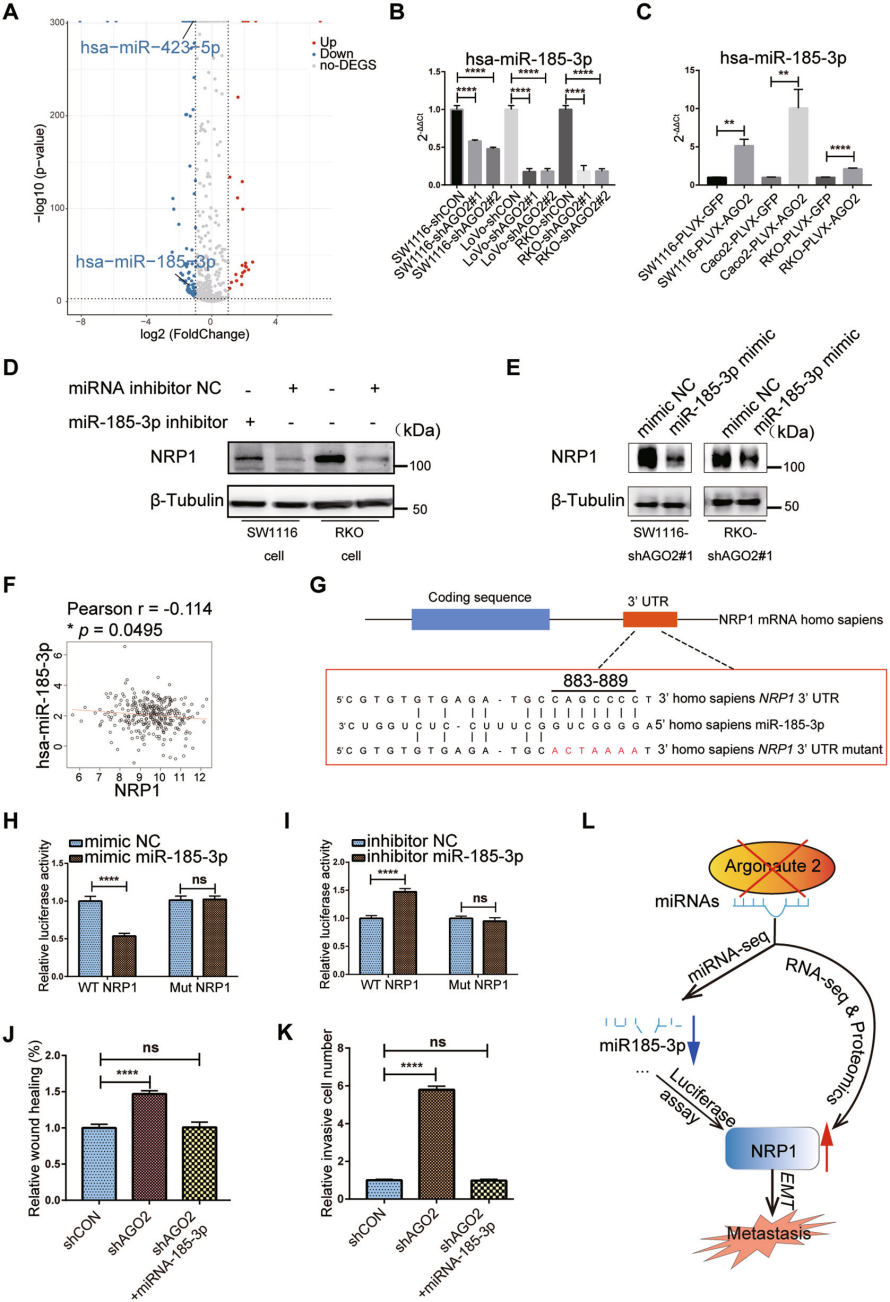
AGO2 knockdown reduces the level of miR-185-3p and NRP1 is a direct target of miR-185-3p. **A** Volcano plot showing miRNAs differentially represented in miRNA-seq expression data upon AGO2 knockdown. **B**, **C** The expression of miR-185-3p was determined by qRT-PCR in CRC cells upon AGO2 knockdown (**B**) or overexpression (**C**). **D** Western blotting analysis of NRP1 in CRC cells treated with miR-185-3p inhibitor or its negative control. **E** Western blotting analysis of NRP1 in SW1116-shAGO2#1 and RKO-shAGO2#1 cells treated with miR-185-3p mimic or its negative control. **F** Pearson correlation analysis between miR-185-3p and NRP1 in CRC tumor tissues. **G** Schematic of the miR-185-3p binding site in the 3′UTR of *NRP1* mRNA and the corresponding mutation. **H**, **I** Luciferase reporter plasmids (NRP1-WT and NRP1-Mut) were co-transfected into 293T cells with miR-185-3p mimic (**H**), miR-185-3p inhibitor (**I**) or the corresponding negative control. Luciferase activity was measured after 48 h. **J**, **K** Statistical analyses showing that migration (**J**) and invasion (**K**) were rescued by the miR-185-3p mimic. **L** Diagram of impaired AGO2/miR-185-3p/NRP1 axis-promoted metastasis in CRC cells. Data are shown as the mean ± SD. *p < 0.05, **p < 0.01, ***p < 0.001, and ****p < 0.0001.

AGO2 is a master regulator of miRNA maturation, stability, and function. Knockdown of AGO2 may lead to reduced numbers of the mature miRNAs that regulate the expression of NRP1. To test this hypothesis, we performed miRNA sequencing of SW1116 AGO2-KD and AGO2-CON cells. Eighty-one miRNAs were significantly reduced, and 24 miRNAs were significantly increased in the AGO2-KD group (Fig. 6A). We found that NRP1 was a potential target gene of miR-185-3p, miR-423-5p and miR-7108-5p in SW1116 cells by using computational methods RNAhybrid, TargetScan, and miRanda. RT-PCR analysis further confirmed that miR-185-3p was significantly reduced in three different AGO2-KD CRC cells and upregulated in AGO2-overexpressing CRC cells (Fig. 6B, C), but the other two miRNAs showed no significant change (Fig. S6A, B). And the expression level of the pre-miR-185-3p was unaltered (Fig. S6C). Kyoto Encyclopedia of Genes and Genomes (KEGG) analysis and pathway enrichment analysis identified target genes significantly enriched in the signal transduction pathway and PI3K-AKT pathway (Fig. S6D, E). Interestingly, NRP1 has been documented to participate in the PI3K-AKT pathway^38^. Western blotting confirmed that the miR-185-3p inhibitor significantly upregulated NRP1 expression in CRC cells (Fig. 6D), and NRP1 protein levels were reduced after transfection with the miR-185-3p mimic in SW1116-shAGO2#1 and RKO-shAGO2#1 cell (Fig. 6E). Furthermore, we found a negative correlation between hsa-miR-185-3p and NRP1 in primary CRC tissues from the TCGA data (Fig. 6F).

There is only one target site for miR-185-3p in the 3′-UTR of *NRP1* (Fig. 6G). Dual luciferase assays showed that the miR-185-3p mimic reduced the luciferase activity of the WT *NRP1* 3′-UTR (Fig. 6H). Consistently, the miR-185-3p inhibitor increased the luciferase activity of the WT *NRP1* 3′-UTR (Fig. 6I). We restored miR-185-3p expression in AGO2-KD cells and found that transfection of the miR-185-3p mimic rescued the phenotypes of the AGO2-KD cells (Fig. 6J, K and Fig. S6F, G). Taken together, these findings suggest that the expression levels of tumor-suppressive miR-185-3p are associated with the intracellular levels of AGO2 and that *NRP1* is a direct target of miR-185-3p in CRC cells.

## Discussion

Impaired miRNA processing by knockdown of Drosha, DGCR8, or Dicer can reduce steady-state miRNA levels and enhance the proliferation and motility of cancer cells^6,39^. In this study, we observed a reduction in AGO2 protein, a core component of the RISC complex, in CRC tumor tissues compared to their normal counterparts. Reduced AGO2 was an independent prognostic factor for DFS and OS and might function as an inhibitor of EMT. Although we found that the expression level of AGO2 had no influence on the proliferation of CRC cells, impaired AGO2 promoted the migration and invasion of CRC cells in vitro and in vivo. These data are consistent with the suppressive function of AGO2 on cell motility demonstrated in multiple cancers^22–26^.

We performed integrated RNA-seq and proteomic analyses and found that AGO2 knockdown led to the upregulation of NRP1, not only at the mRNA level but also at the protein level in multiple CRC cells. NRP1 is well recognized as a key molecule in the process of cancer invasion and metastasis, and its overexpression has been detected in various metastatic tumors^35,36,40^. Importantly, EMT in CRC might be NRP1-dependent^41^. Consistently, we found that the knockdown of NRP1 also exhibited strong inhibitory effects on CRC cell migration, invasion, and metastasis. EMT-associated protein N-cadherin, vimentin, and E-cadherin levels were altered upon AGO2 or AGO2/NRP1 knockdown. However, the underlying mechanism of how EMT biomarkers are positively controlled by NRP1 still needs further investigation. It might involve the PI3K-AKT pathway.

AGO2 contributes to the biogenesis of miRNAs and binds to mature miRNAs to protect them from degradation^10–13^. miRNA downregulation is frequently observed in metastatic tumors^42^, and miRNAs collectively function to suppress cell migration and invasion^43^. In this study, we found that the impairment of AGO2 resulted in decreased miRNA expression, especially miR-185-3p. Previous studies have profiled miRNAs and shown that miR-185-3p is downregulated in CRC^44,45^, and miR-185-3p has been demonstrated to be a tumor suppressor in many types of cancer, such as nasopharyngeal carcinoma, breast cancer, and CRC^46–49^. However, we did not find any change in the expression of pre-miR-185-3p, which indicated that the AGO2 protein might affect the processing of miR-185-3p. The restoration of the miR-185-3p mimic in AGO2-KD cells rescued the effects of AGO2 knockdown indicating that AGO proteins are initially made in excess relative to the miRNAs, and that the other AGO proteins could compensate for the function of AGO2 in the RISC complex^50^. Furthermore, we found that *NRP1* was the direct target of miR-185-3p. The knockdown of AGO2 led to an increase in NRP1 at both the mRNA and protein levels, which is consistent with studies of transcriptomics, proteomics, and ribosome profiling, revealing that target mRNA degradation by miRNA is more widespread than translational repression by miRNA^51,52^.

In summary, we found that the impairment of AGO2 led to decreased miR-185-3p, which is potentially due to a loss of protection from degradation, and that miR-185-3p could regulate cell migration and invasion by directly targeting *NRP1* in CRC. We identified a novel AGO2/miR-185-3p/NRP1 regulatory axis that modulates EMT and the metastatic capability of CRC cells, which might represent a regulatory process for cancer cell migration and metastasis in general (Fig. 6L). Furthermore, there might be important translational implications of our findings, as the delivery of some mimics, such as a miR34 mimic, have promising tumor-suppressing effects^53^. The miR-185-3p mimic or targeted inhibition of *NRP1* might also be an effective therapeutic approach to block CRC metastasis.

## Materials and methods

### Tissue specimens

CRC tissues, matched normal colon tissues, and metastatic foci were collected at the Shanghai General Hospital from 2008 to 2013 and used in this study. Prior to surgery, no patients received radiotherapy, chemotherapy, or other related neoadjuvant therapies. This research was approved by the Ethics Committee of Shanghai General Hospital, and informed consent was obtained from all patients enrolled in the study.

### Cell culture

The Caco2, HCT116, HT29, LoVo, RKO, SW1116, SW480, NCM460, and HEK 293T cell lines were purchased from the Type Culture Collection of the Chinese Academy of Science (Shanghai, China). Cell stocks were created within five passages, and all experiments were completed within ten passages. A new aliquot of the same batch of cells was thawed every 1–2 months to restart the passaging. These cells were cultured in Dulbecco’s modified Eagle medium supplemented with 10% fetal bovine serum (Gibco, Grand Island, NY, USA) and 1% penicillin/streptomycin. All cells were maintained at 37 °C in a humidified atmosphere containing 5% CO_2_ and confirmed to be free of mycoplasma contamination by PCR.

### Multiplex fluorescent immunohistochemistry (IHC) of CRC tissue microarrays (TMA) and multispectral imaging and Scoring multispectral images

Multiplex staining was performed using an OpalTM 4-Color Manual IHC Kit (PerkinElmer, Hopkinton, MA, USA) according to the manufacturer’s protocol. Consecutive primary antibody staining rounds included the following antibodies at the indicated dilutions: AGO2 (1:500), vimentin (1:2000), and E-cadherin (1:500). The detailed information was refereed to published report^30^. Slides were examined using a VECTRA 3.0 Automated Quantitative Pathology Imaging System (PerkinElmer). InForm image analysis software (PerkinElmer) was used to analyse expression of each marker, unmix multispectral images with high contrast and accuracy, and batch analysis of multispectral images. The *H*-scores of AGO2, E-cadherin, and vimentin were listed in supplementary data

### Cell transfection

Plasmids were transfected using Lipofectamine 2000 (#11668019, Invitrogen; Thermo Fisher Scientific). miRNA mimics and inhibitors were transfected using NanoEnter® Transfection Reagent (C510000, New Cell & Molecular Biotech). The sequences of shRNA targeting AGO2, NRP1, miRNA mimics, and inhibitors were listed in Supplementary Table S3.

### Western blot analysis

Western blot assay was performed as described previously^54^. And the primary antibodies we used were listed in Supplementary Table S4.

### Quantitative real-time PCR analysis

Total RNA was extracted using Trizol reagent (Invitrogen) according to the manufacturer’s instructions. cDNA was synthesized using SuperScript VILO™ cDNA Synthesis Kit (Invitrogen). Quantitative real-time PCR analysis was performed using SYBR Select Master Mix (Applied Biosystems) in a real-time PCR machine (QuantStudio 6 Flex Real-Time PCR system; Thermo Fisher Scientific).

For miRNA, total RNA was extracted using the miRNeasy Micro Kit (Qiagen) according to manufacturer’s instructions. The miScript II RT Kit was used to obtain the reverse transcriptions of miRNAs, and the miScript SYBR Green PCR Kit (Qiagen, Germany) was used to perform quantitative PCR. Precursor miRNAs were quantified using TaqMan Pre-miRNA assays. Sno202 and U6 were used as endogenous controls for Pre-miRNA and miRNA expression in analysis, respectively. Primers were listed in Supplementary Tables S5, 6.

### Cell proliferation and colony formation assays

CCK8 cell proliferation assay and colony formation assay were performed as described previously^54^.

### Flow cytometry analysis of colon cancer cells apoptosis

5-FU induced apoptosis assay was performed as described previously^54^. Cells were seeded in a 6-well plate 24 h before treatment with 50 μg/ml 5-FU for 24 h, then harvested, stained and analysed by a Fortessa cytometer (BD Biosciences, San Jose, CA, USA).

### Wound healing assay

Cells were seeded into 6-well plates with growth medium. Near 80% confluency, the cells were wounded by scraping the monolayers with a sterile 200 μl plastic tip. Images of cells migration into the wound area were captured at different time points.

### Cell migration and invasion assay

Transwell migration and invasion assays were performed as described previously^55^. Finally, the transwell membranes were excised and mounted on a standard microscope slide. And cell numbers were determined from 2 to 3 random fields visualized at ×10 magnification.

### Tumorigenesis in nude mice

All animal experiments were approved by the Animal Care and Use Committee at The Shanghai Jiao Tong University School of Medicine in accordance with China’s Ministry of Health national guidelines for housing and care of laboratory animals. Cells were injected into the flank regions of 6-week-old female BALB/c nude mice purchased from the Institute of Zoology, Shanghai Jiao Tong University School of Medicine, Shanghai, as described previously^54^. Four weeks later, the mice were sacrificed, and the tumors were harvested, photographed, and prepared for haematoxylin and eosin (H&E) staining.

### In vivo metastasis experiments

For the metastasis experiments, 2 × 10^6^ CRC cells were injected into the spleens of female BALB/c nude mice (*n* = 6 for each group). Briefly, mice were anaesthetized via intraperitoneal (IP) injection of pelltobarbitalum natricum, and following abdominal incision, 2 × 10^6^ CRC cells in 200 µl were injected into the exteriorized spleen using an insulin syringe. Five minutes after the injection, the spleen blood vessels were ligated, the spleen was removed, and the abdominal wound was closed with staples. After 6–8 weeks, the mice were sacrificed, their livers were removed, and the tumor nodules were prepared for H&E staining. For each sample, all micro-metastases were counted under a light microscope at ×10 magnification. Three sections were counted per mouse sample at 50 μm intervals, and the results were averaged.

### RNA-seq and data analysis

Total RNA from SW1116-shAGO2#1 and SW1116-shCON cells was purified using an Invitrogen PureLink mini kit. Libraries were prepared using the Illumina TruSeq RNA Sample Prep Kit as described by the manufacturer’s protocol. Samples were sequenced on the Illumina HiSeq 2000. Adapters and low-quality sequences were removed to obtain clean data by OAPnuke. The clean data were mapped to the human genome (hg38) using HISAT2 assembled by StringTie. Differentially expressed genes were calculated by the Ballgown package in R. Genes with RPKM (reads per kilobase per million mapped reads) values less than 1 in all samples were removed. Of the 17,007 genes identified in all samples, 245 (fold change ≥ 2 and *p*-value ≤ 0.001) genes were differentially expressed in SW1116-shAGO2#1 cell compared with their SW1116-shCON counterpart, which were listed in supplementary data.

### miRNA-seq and data analysis

Total RNA, including miRNA of SW1116-shAGO2#1 and SW1116-shCON cells, was extracted using the miRNeasy Micro Kit (Qiagen). High-throughput sequencing of miRNA was processed according to the TruSeq Small RNA Sample Preparation Guide (Illumina). Briefly, the total RNA was run on an agarose gel, and the band corresponding to the size of miRNAs was extracted for further processing. Sequencing adapters were ligated to the size-selected RNA molecules, and reverse transcription was carried out to obtain cDNA libraries, which were subsequently sequenced by Illumina HiSeq2500. After sequencing, adapters and low-quality sequences were removed from the obtained raw reads. Fragments of 15–30 nt were reserved for subsequent analysis and mapped to the reference genome using Bowtie2. The clean data were then compared to the known miRNA in miRBase using Bowtie2. miRDeep2 was used to predict novel miRNA. miRNA expression levels were normalized to RPM (reads per million). Read counts less than 50 in all samples were removed. Differentially expressed miRNAs were calculated using DEGseq. A fold change ≥ 2 and *Q*-value ≤ 0.001 were considered differentially expressed miRNAs, and they were listed in supplementary data. RNAhybrid, TargetScan and miRanda were used to predict miRNA targets.

### The miRNA-seq and RNA-seq level correlation analysis

Three data of CRC patient were downloaded from TCGA database. Data platforms for miRNAseq and RNAseq were BDGSC_UNC_HSmiR_miRNAseq and UNC_IlluminaHiSeq_RNAseq, respectively. There were 188 CRC samples were obtained. Pearson correlation was calculated between hsa-miR-185-3p and *NRP1*. Negative correlations of expression were found between hsa-miR-185-3p and *NRP1* (correlation value = −0.18, *p*-value = 1.3e−2).

### Preparation of lysates and LC-MS/MS quantitative proteomic analysis

Cytosolic and nuclear proteins from SW1116-shAGO2#1 and SW1116-shCON cell samples were extracted from ~6 × 10^6^ cells. The detailed information was referred to the published report^54^. The LC-MS/MS datasets were analysed with MaxQuant. MS/MS spectra were searched against SwissProt human databases, which comprised 20,317 entries. Peptide-spectrum matches from all datasets were assembled into proteins, and 5849 proteins were finally obtained. A paired *t*-test was used to calculate differentially expressed proteins, with a *p*-value less than 0.05 and fold change ≥ 1.3. And the differential proteins were listed in supplementary data.

### Plasmids and luciferase reporter assay

The plasmids of human wildtype NRP1 3′-UTR and miR185-3p-binding site mutated NRP1 3′-UTR were constructed as described previously^56^. To determine the miR-185-3p/*NRP1* mRNA interaction, HEK 293T cells were seeded in 24-well plates, and reporter gene constructs were co-transfected into cells with miR-185-3p mimic or inhibitor for 48 h. An expression vector encoding Renilla luciferase at a 1:50 ratio was included during the transfection. And the detailed information was same as described previously^56^.

The PCR primer sequences for DNA construction and mutagenesis were as follows: *NRP1* 3′-UTR, forward 5′-CCGCTCGAGATAGGCAAAGAAGGATTAGGC-3′, reverse 5′-GCTCTAGAAGACTTTGGAGGGCAAAGTTAGGAATTTCCC-3′;

*NRP1* 3′-UTR-mut, forward 5′-GTGAGATGCACTAAAATCCGGGCAGGCAAGGGCT-3′, reverse 5′-CCTGCCCGGATTTTAGTGCATCTCACACGAAAATAAAC-3′.

### Statistical analysis

Statistical analysis was performed using SPSS 23.0 software (IBM SPSS, USA) and GraphPad Prism 7.0 software (GraphPad Software, USA) for Windows. All data are presented as the mean ± SD unless otherwise specified. Correlations of AGO2 with E-cadherin and vimentin expression levels were performed by calculating the Pearson correlation coefficient. Chi-squared tests were applied to analyse the relationship between AGO2 expression and clinicopathological status. Groups of discrete variables were compared by means of the Mann–Whitney *U*-test or Kruskal–Wallis nonparametric analysis of variance. Kaplan–Meier curves were used for survival analysis, and differences among patient subgroups were calculated using log-rank tests. Univariate and multivariate Cox regression analyses were used to identify the prognostic factors that influenced survival. All experiments with cell cultures were performed independently at least three times and in triplicate each time. *p-*values < 0.05 were considered statistically significant.

## Supplementary information

Supplementary tables S1-S6

Supplementary Figure S1

Supplementary Figure S2

Supplementary Figure S3

Supplementary Figure S4

Supplementary Figure S5

Supplementary Figure S6

Supplementary Figures legends

Supplementary Data

## Data Availability

The mass spectrometry proteomics data have been deposited to the ProteomeXchange Consortium (http://proteomecentral.proteomexchange.org) via the iProX partner repository^57^ with the dataset identifier PXD024171. Raw sequencing data of miRNA-seq is available in Sequence Read Archive (SRA) database with accession number SUB9049740.
