# Supplementary material for: Impaired AGO2/miR-185-3p/NRP1 axis promotes colorectal cancer metastasis
Source: Cell Death Dis. 2021 Apr 12;12(4):390. doi: 10.1038/s41419-021-03672-1 (PMC8042018; doi:10.1038/s41419-021-03672-1)
Supplement: Supplementary file 1 — Supplementary tables S1-S6 [file 41419_2021_3672_MOESM1_ESM.docx]

**SUPPLEMENTAL TABLES:**

**Supplementary Table S1. Univariate analysis of clinicopathologic parameters associate with disease-free survival and overall survival.**

| **Univariate analysis**  **Variables** | **Disease-free survival** | | | **Overall survival** | | |
| --- | --- | --- | --- | --- | --- | --- |
|  | **HR** | **95% CI** | ***P*** | **HR** | **95% CI** | ***P*** |
| **Age (years, ≤65 vs. >65)** | 0.755 | 0.465-1.225 | 0.225 | 1.286 | 0.920-1.796 | 0.141 |
| **Gender (Female vs. Male)** | 1.196 | 0.735-1.944 | 0.471 | 0.990 | 0.721-1.360 | 0.951 |
| **Tumor size (≤5cm vs. >5cm)** | 1.204 | 0.729-1.988 | 0.468 | 1.157 | 0.834-1.606 | 0.384 |
| **pT (T3-4 vs T1-2)** | 4.983 | 2.000-12.414 | **<0.001** | 3.386 | 2.067-5.545 | **<0.001** |
| **pN (N1-2 vs No)** | 6.556 | 3.501-12.279 | **<0.001** | 3.178 | 2.264-4.462 | **<0.001** |
| **pM (M1 vs M0)** | 9.475 | 5.708-15.727 | **<0.001** | 4.766 | 3.290-6.904 | **<0.001** |
| **AJCC stage (III-IV vs I-II)** | 7.876 | 4.010-15.468 | **<0.001** | 3.492 | 2.469-4.941 | **<0.001** |
| **Tumor grade**  **(Well & Moderate vs Poor)** | 3.592 | 2.199-5.869 | **<0.001** | 2.714 | 1.937-3.802 | **<0.001** |
| **AGO2 expression** | 0.441 | 0.236-0.825 | **0.010** | 0.627 | 0.433-0.908 | **0.014** |

HR hazard ratio, CI confidence interval;

*P* < 0.05 was considered significant

**Supplementary Table S2. Multivariate analysis of clinicopathologic parameters associate with disease-free survival and overall survival.**

| **Multivariate analysis**  **Variables** | **Recurrence-free survival** | | | **Overall survival** | | |
| --- | --- | --- | --- | --- | --- | --- |
|  | **HR** | **95% CI** | ***P*** | **HR** | **95% CI** | ***P*** |
| **Age (years, ≤65 vs. >65)** | 0.727 | 0.446-1.185 | 0.201 | 1.302 | 0.931-1.821 | 0.124 |
| **Gender (Female vs. Male)** | 1.098 | 0.671-1.798 | 0.709 | 0.914 | 0.663-1.259 | 0.581 |
| **Tumor size (≤5cm vs. >5cm)** | 1.207 | 0.730-1.998 | 0.463 | 1.185 | 0.853-1.645 | 0.311 |
| **Tumor grade**  **(Well & Moderate vs Poor)** | 3.513 | 2.143-5.760 | **<0.001** | 2.728 | 1.942-3.832 | **<0.001** |
| **AGO2 expression** | 2.200 | 1.176-4.116 | **0.014** | 1.559 | 1.076-2.258 | **0.019** |

HR hazard ratio, CI confidence interval;

*P* < 0.05 was considered significant

**Supplementary Table S3**: **The sequences of shRNA targeting AGO2 and NRP1, miRNA mimics and inhibitors.**

| **Target gene** | **Sequence** |
| --- | --- |
| **shAGO2#1** | ACAGATTCCCAAAGGGTAAAG |
| **shAGO2#2** | CGTCCGTGAATTTGGAATCAT |
| **shNRP1** | GCAACGATAAATGTGGCGATA |
| **miR-185-3p mimic** | AGGGGCUGGCUUUCCUCUGGUC |
| **miR-185-3p inhibitor** | GACCAGAGGAAAGCCAGCCCCU |
| **mimic Negative Control** | UUUGUACUACACAAAAGUACUG |
| **inhibitor Negative Control** | CAGUACUUUUGUGUAGUACAAA |

**Supplementary Table S4. Primary and neutralizing antibodies used in this study.**

| **Antibodies** | **Dilution** | **Clone, source** | **Company** |
| --- | --- | --- | --- |
| **AGO2** | 1:500 | Rabbit polyclonal | ab32381, Abcam |
| **NRP1** | 1:1000 | Rabbit polyclonal | Ab81321, Abcam |
| **HMGA2** | 1:1000 | Rabbit polyclonal | Ab97276, Abcam |
| **CPA4** | 1:500 | Rabbit polyclonal | Ab81543, Abcam |
| **CDK6** | 1:1000 | Mouse monoclonal | CS3116, Cell Signaling |
| **IGF2B2** | 1:1000 | Rabbit monoclonal | CS14672, Cell Signaling |
| **N-Cadherin** | 1:1000 | Rabbit monoclonal | CS13116, Cell Signaling |
| **E-Cadherin** | 1:1000 | Rabbit monoclonal | CS3195, Cell Signaling |
| **Vimentin** | 1:1000 | Rabbit monoclonal | CS5741, Cell Signaling |
| **SNAIL1** | 1:1000 | Rabbit polyclonal | GTX125918, GeneTex |
| **TWIST1/2** | 1:1000 | Rabbit polyclonal | GTX127310, GeneTex |
| **ZEB1** | 1:1000 | Rabbit polyclonal | GTX105278, GeneTex |
| **β-Tubulin** | 1:5000 | Mouse monoclonal | KM9007T, SUNGENE BIOTEX |
| **NRP1** | 5 ug/mL | Sheep polyclonal | AF3780, R&D |
| **IgG** | 5 ug/mL | Sheep polyclonal | 5-001-A, R&D |

**Supplementary Table S5**: **Coding genes and luciferase reporter plasmids Primers.**

| **Gene** | **Forward Primer** | **Reverse Primer** |
| --- | --- | --- |
| **AGO2** | CGTGCCTGCTGGAATGTTTC | CCATCCGTGAGGCCTGTATC |
| **β-ACTIN** | AAGGTGACAGCAGTCGGTT | TGTGTGGACTTGGGAGAGG |
| **NRP1** | CGTTCTTCCGAAATGTTGATTG | TCCCCAGAAATATTTGGTTTAAAGG |
| **HMGA2** | AGTCCCTCTAAAGCAGCTCA | GTCCTCTTCGGCAGACTCTT |
| **CPA4** | TGCAACACAATGAAGGGCAAG | CGGCAATGTTGTCCATCTCG |
| **AMIGO2** | CTCAGAGGCGACCATAATGTC | TGTTTATTTTGCAGACCACACAC |
| **N-Cadherin** | CATCATCCTGCTTATCCTTGTG | CATAGTCCTGGTCTTCTTCTCC |
| **E-Cadherin** | GTCCTGGGCAGACTGAATTT | GACCAAGAAATGGATCTGTGG |
| **Vimentin** | GAGAACTTTGCCGTTGAAGC | GCTTCCTGTAGGTGGCAATC |
| **SNAIL1** | ATGCCGCGCTCTTTCCTCGTC | AGCAGGTGGGCCTGGTCGTAG |
| **TWIST1** | GTCCGCAGTCTTACGAGGAG | GCTTGAGGGTCTGAATCTTGCT |
| **ZEB1** | AAGAATTCACAGTGGAGAGAAGCCA | CGTTTCTTGCAGTTTGGGCATT |

**Supplementary Table S6**: **miRNAs primers.**

| **miRNA** | **Stem-loop RT Primer** | **Forward Primer** | **Reverse Primer** |
| --- | --- | --- | --- |
| **miR-185-3p** | GTCGTATCCAGTGCGTGTCGTGGAGTCGGCAATTGCACTGGATACGACGACCAG | GATGGGAGGGGCTGGCTTTCCTCT | CAGTGCGTGTCGTGGAGTC |
| **miR-423-5p** | GTCGTATCCAGTGCGTGTCGTGGAGTCGGCAATTGCACTGGATACGACAAAGTC | GATGGGTGAGGGGCAGAGAGCGAGA | TGTAAACATCCTCGACTGGAAG |
| **miR-7108-5p** | GTCGTATCCAGTGCGTGTCGTGGAGTCGGCAATTGCACTGGATACGACCCACCC | GATGGGGTGTGGCCGGCAGGCGG | CAGTGCGTGTCGTGGAGTC |
| **U6** | GTCGTATCCAGTGCAGGGTCCGAGGTGCACTGGATACGACAAAATATGG | TGCGGGTGCTCGCTTCGGCAGC | CCAGTGCAGGGTCCGAGGT |
