# Supplementary figures and images for: Impaired AGO2/miR-185-3p/NRP1 axis promotes colorectal cancer metastasis
Source: Cell Death Dis. 2021 Apr 12;12(4):390. doi: 10.1038/s41419-021-03672-1 (PMC8042018; doi:10.1038/s41419-021-03672-1)

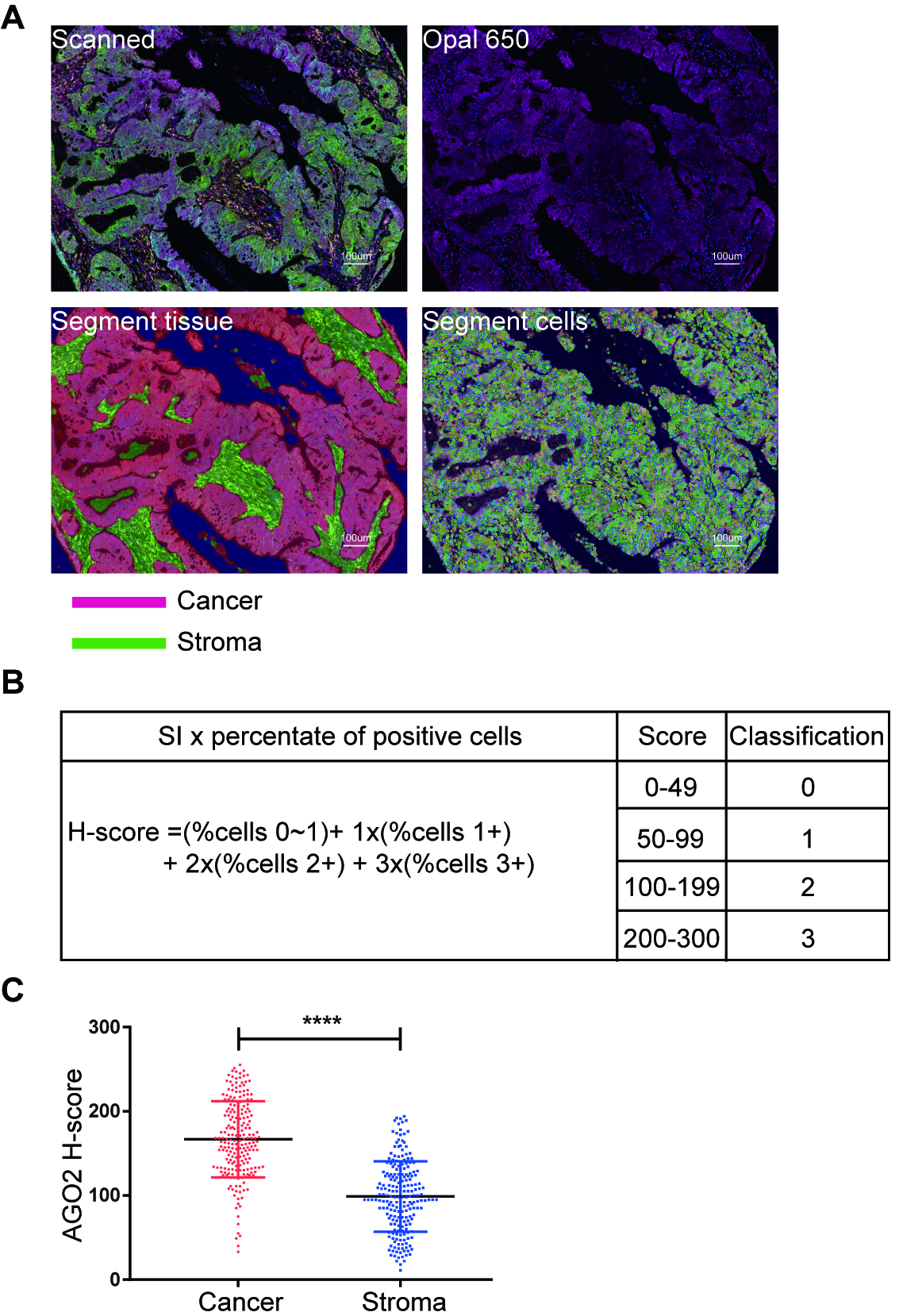

Supplement: Supplementary file 2 — Supplementary Figure S1 [file 41419_2021_3672_MOESM2_ESM.tif]

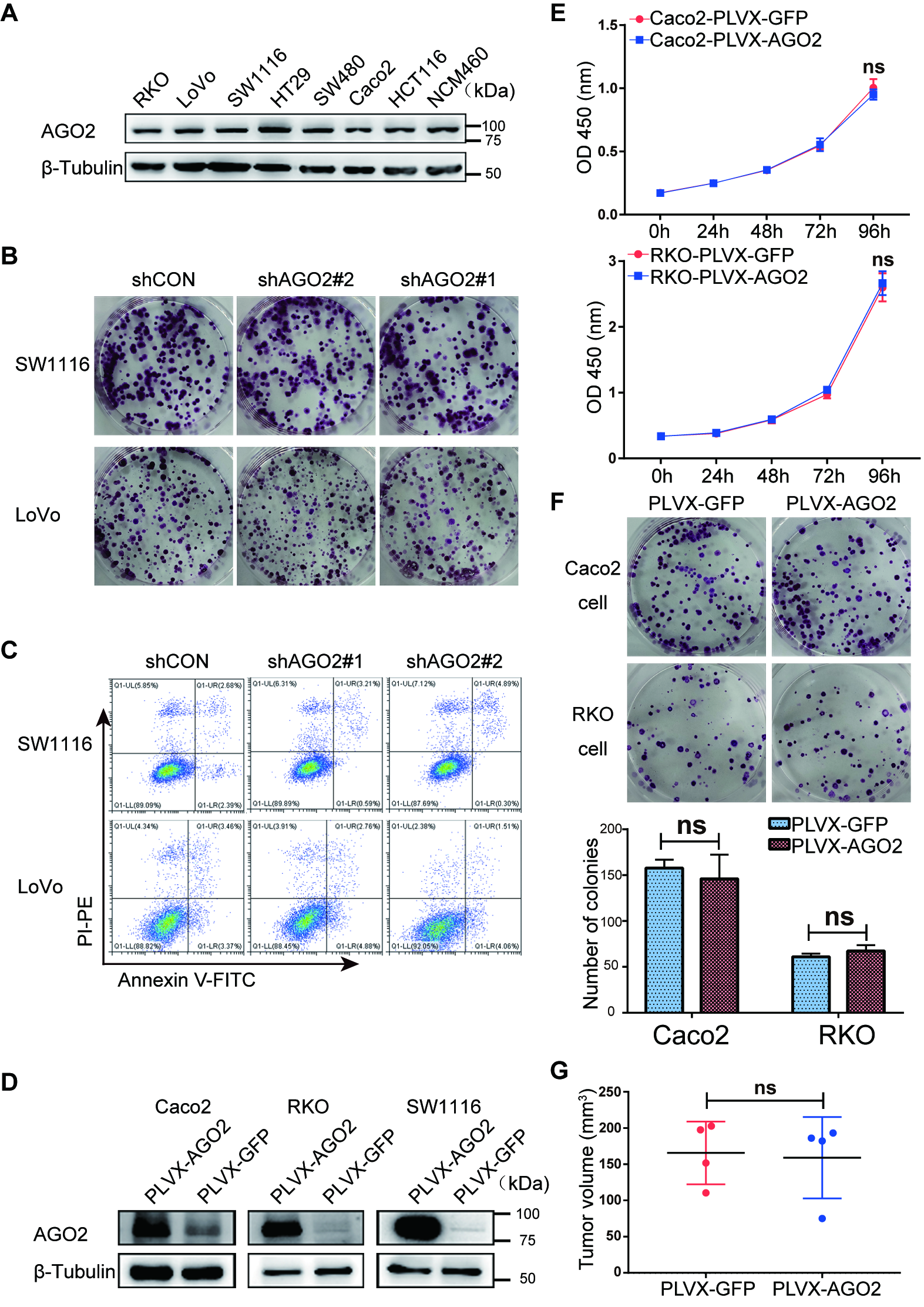

Supplement: Supplementary file 3 — Supplementary Figure S2 [file 41419_2021_3672_MOESM3_ESM.tif]

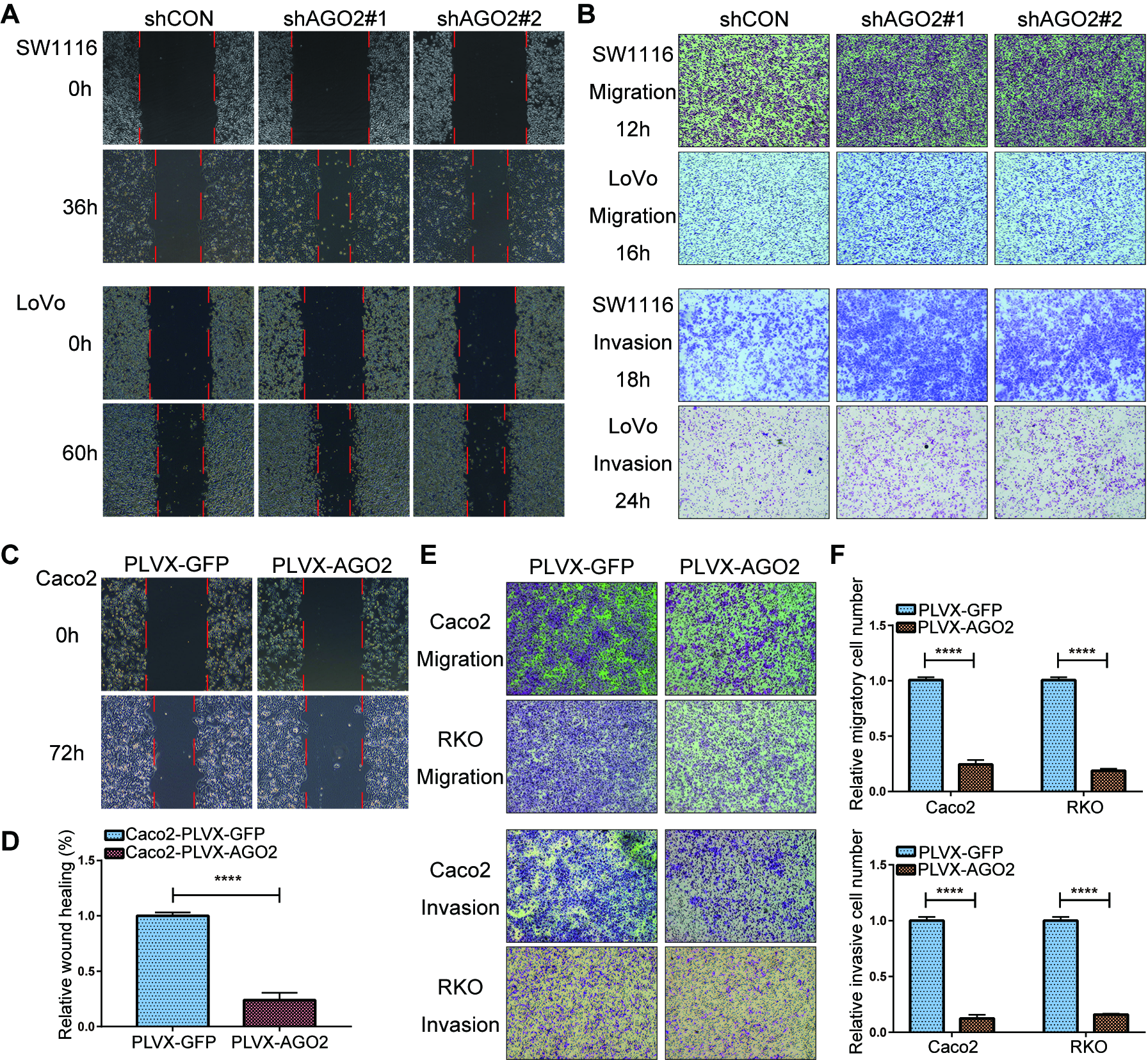

Supplement: Supplementary file 4 — Supplementary Figure S3 [file 41419_2021_3672_MOESM4_ESM.tif]

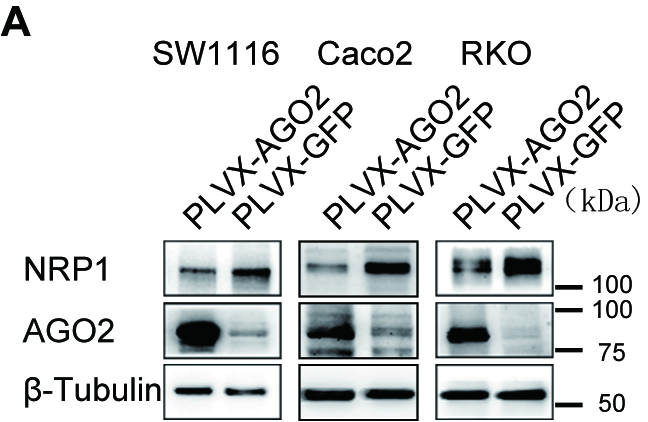

Supplement: Supplementary file 5 — Supplementary Figure S4 [file 41419_2021_3672_MOESM5_ESM.tif]

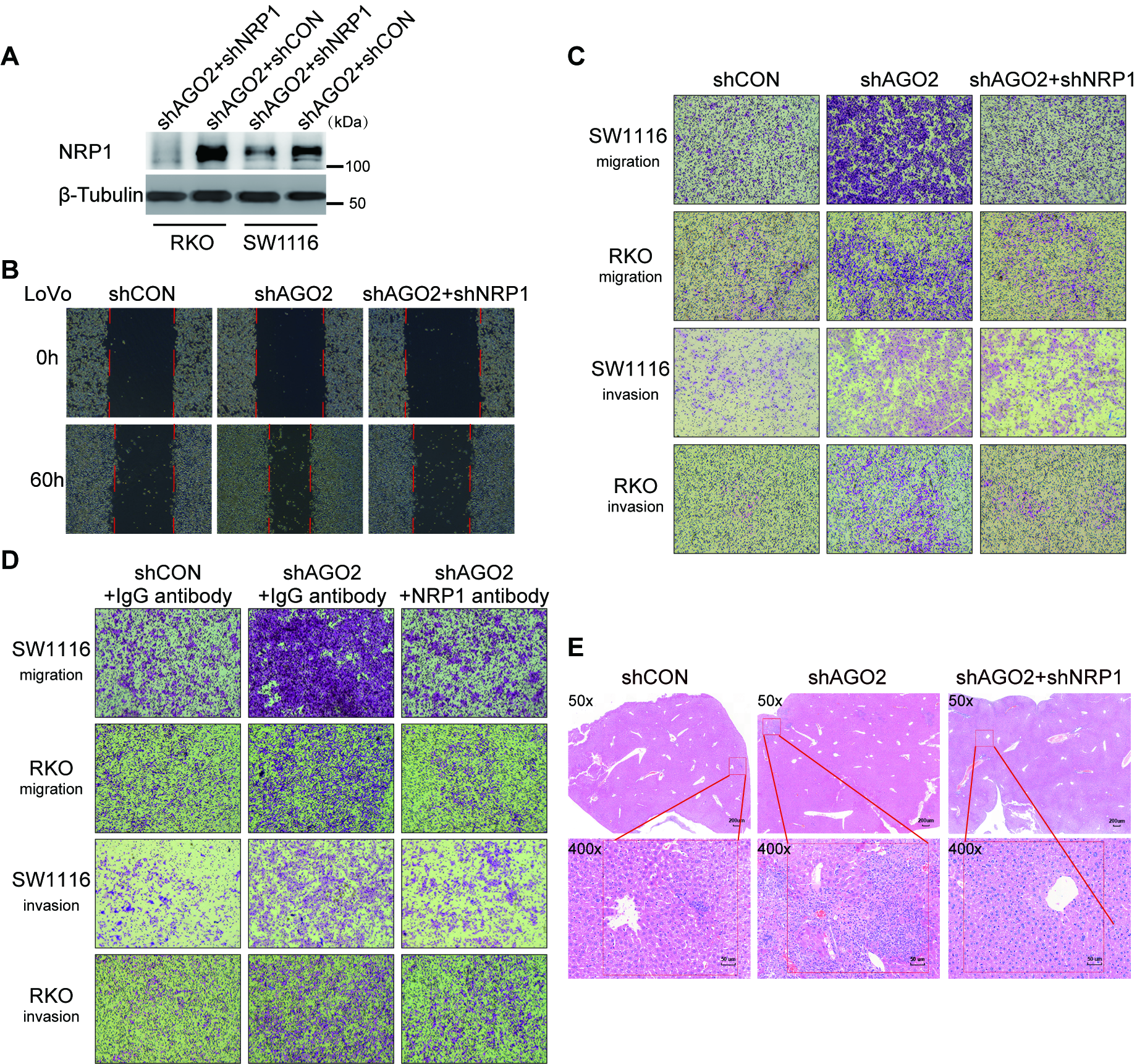

Supplement: Supplementary file 6 — Supplementary Figure S5 [file 41419_2021_3672_MOESM6_ESM.tif]

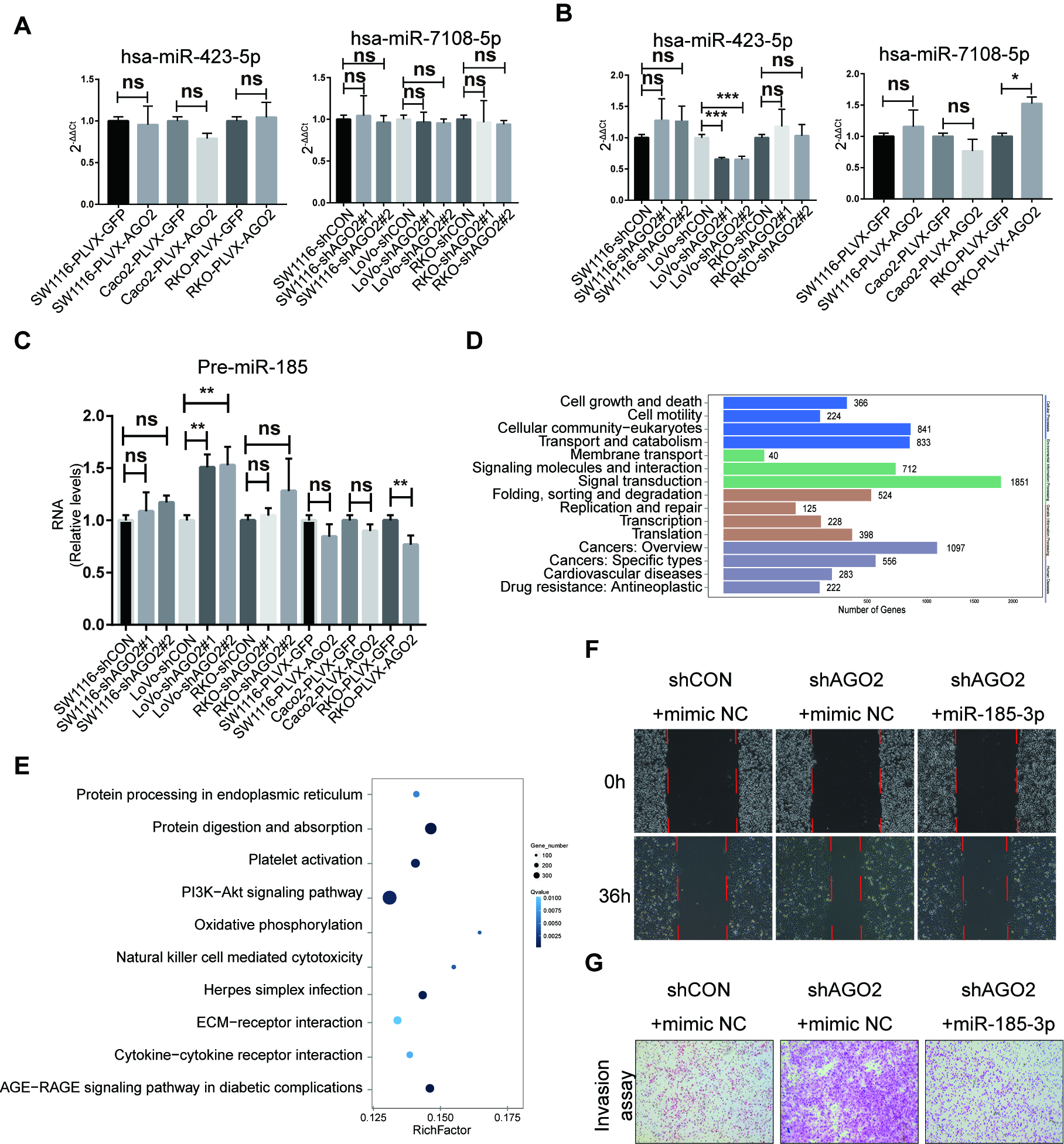

Supplement: Supplementary file 7 — Supplementary Figure S6 [file 41419_2021_3672_MOESM7_ESM.tif]
