# Supplementary material for: Impaired AGO2/miR-185-3p/NRP1 axis promotes colorectal cancer metastasis
Source: Cell Death Dis. 2021 Apr 12;12(4):390. doi: 10.1038/s41419-021-03672-1 (PMC8042018; doi:10.1038/s41419-021-03672-1)
Supplement: Supplementary file 8 — Supplementary Figures legends [file 41419_2021_3672_MOESM8_ESM.docx]

**Supplementary Figure Legends**

**Supplementary Figure S1. A.** Pipeline for tissues and cells segment by InForm software. The image of raw multiple fluorescent channels merged was separated into two fluorescent channels (Opal650 and DAPI). Segment tissues were divided into cancer and stroma based on the fluorescent signal intensity of DAPI. **B.** H-score was automatic calculated four levels (0~1, 1~2, 2~3, 3~) and percentage positivity of cells with each bin of stained protein by InForm software. **C.** Comparing levels of AGO2 protein based on H-Score in cancer tissue and stroma tissue of patients with CRC. Data are shown as the mean ± SD. *****p* < 0.0001.

**Supplementary Figure S2. A.** Representative immunoblots for AGO2 expression in different kinds of CRC cell lines and normal colon cell line NCM460. **B.** Images of colony formation assays of SW1116-shAGO2 and LoVo-shAGO2 in comparison with corresponding controls. **C.** Flow images of early and late apoptotic cells of SW1116-shAGO2 and LoVo-shAGO2 in comparison with corresponding controls. **D.** Representative immunoblots for AGO2 expression in CRC cell lines stably transfected with AGO2 expression vector or PLVX-control vector. **E.** CCK8 proliferation assay of Caco2 (upper) and RKO (bottom) CRC cells. Two CRC cells were stably transfected with AGO2 expression vectors (PLVX-AGO2) in comparison with corresponding empty/control (PLVX-GFP) vectors. **F.** Images (upper) and statistical analysis (bottom) of colony formation assays of Caco2 and RKO cell enforced expression of AGO2 in comparison with corresponding controls. **G.** Statistics of tumors isolated from subcutaneous xenografts in female BALB/c nude mice injected with Caco2-PLVX-AGO2 cells and Caco2-PLVX-GFP control, (n = 4 mice per group). Data are shown as the mean ± SD. ns, *p* > 0.05, two-tailed Student’s t-test.

**Supplementary Figure S3. A.** Representative image of the migratory potential of AGO2 knockdown SW1116 (upper) or LoVo (bottom) cells and the respective control cells was analyzed in a wound-healing assay for 0, 36, and 60 h. at 10x magnification. **B.** Representative image of the migratory potential (upper) and invasive potential (bottom) of AGO2-knockdown SW1116 or LoVo cells and the respective control cells were analyzed by the Transwell cell migration and matrigel invasion assay. at 10x magnification. **C.** Representative image of the migratory potential of AGO2 overexpression Caco2 cells and the control cells was analyzed in a wound-healing assay for 0 and 72 h. at 10x magnification. **D.** Statistical analyses of cell migration were determined by wound healing assay upon AGO2-overexpression. **E.** Representative image of the migratory potential (upper) and invasive potential (bottom) of AGO2-overexpression Caco2 or RKO and the respective control cells were analyzed by the Transwell cell migration and matrigel invasion assay. at 10x magnification. **F.** Quantification of the Transwell assay results. All experiments were performed in triplicate and results were presented as the mean ± SD. *****p* < 0.0001.

**Supplementary Figure S4. A.** Western blot analysis of AGO2’s upregulation on the expression of pro-tumorigenesis gene NRP1 in CRC cells.

**Supplementary Figure S5. A.** Representative immunoblots for NRP1 expression in CRC shAGO2 cells stably transfected with shNRP1 or shCON. **B.** Representative image of the migratory potential of AGO2 knockdown, AGO2 NRP1 double knockdown and the empty control LoVo cells was analyzed in a wound-healing assay for 0h and 60 h. at 10x magnification. **C.** Representative image of the migratory potential (upper) and invasive potential (bottom) of AGO2 knockdown and AGO2 NRP1 double knockdown SW1116 and RKO cells and the empty control cells were analyzed by the Transwell cell migration and matrigel invasion assay. at 10x magnification. **D.** Representative image of the migratory potential (upper) and invasive potential (bottom) of AGO2 knockdown SW1116 and RKO cells treated with neutralizing anti-NRP1 antibody or IgG antibody were analyzed by the Transwell cell migration and matrigel invasion assay. at 10x magnification. **E.** Representative H&E-stained images presented to show metastatic nodules in the liver (scale bar, 50 μm and 200 μm). Female BALB/c nude mice were injected via spleen with the SW1116 cells that knockdown AGO2 (shAGO2), double knockdown AGO2 and NRP1 (shAGO2+shNRP1) and the empty control (shCON) cells, (n = 6 mice per group).

**Supplementary Figure S6. A.** Relative expression levels of miR-423-5p and miR-7108-5p in CRC cell lines stably transfected with shAGO2 or shCON. **B.** Relative expression levels of miR-423-5p and miR-7108-5p in CRC cell lines stably transfected with AGO2 expression vector or PLVX-GFP control vector. **C.** Relative levels of pre-miR-185 in CRC stable transfected cell lines silenced or upregulated for AGO2, analyzed by RT-qPCR. **D.** KEGG pathways analysis of changed genes showing the significant pathways in AGO2-knockdown cells. **E.** Target pathway enrichment analysis of target genes differentially represented in miRNA-seq data from SW1116-shAGO2#1 versus SW1116-shCON cell lysis. **F.** Representative image of the migratory potential of AGO2 knockdown and the empty control SW1116 cell treated with mimic miR-185-3p was analyzed in a wound-healing assay for 0h and 60 h. at 10x magnification. **G.** Representative image of the invasive potential of AGO2 knockdown and the empty control SW1116 cell treated with mimic miR-185-3p was analyzed by the Transwell matrigel invasion assay. at 10x magnification. Data are shown as the mean ± SD. ns, no statistical significance (*p* > 0.05), **p* < 0.05, ***p* < 0.01, ****p* < 0.001, and *****p* < 0.0001.
