# Supplementary material for: Impaired AGO2/miR-185-3p/NRP1 axis promotes colorectal cancer metastasis
Source: Cell Death Dis. 2021 Apr 12;12(4):390. doi: 10.1038/s41419-021-03672-1 (PMC8042018; doi:10.1038/s41419-021-03672-1)
Supplement: Supplementary file 9 — Supplementary Data [file 41419_2021_3672_MOESM9_ESM.pdf]

H-scores of AGO2 in cancer, stroma and normal tissues					
Patient ID	AGO2 in Normal	AGO2 in Cancer	AGO2 in Stroma		
D15A1995	94	87	37		
D15A1996	147	142	138		
D15A1997	192	66	99		
D15A1998	155	141	56		
D15A1999	181	131	92		
D15A2000	174	168	95		
D15A2001	206	192	144		
D15A2002	152	131	29		
D15A2003	246	172	40		
D15A2004	187	153	105		
D15A2005	230	138	158		
D15A2006	229	154	117		
D15A2007	187	40	98		
D15A2008	227	206	69		
D15A2009	128	126	22		
D15A2010	164	154	26		
D15A2011	210	180	28		
D15A2012	219	200	154		
D15A2013	200	195	70		
D15A2014	231	212	75		
D15A2015	69	55	54		
D15A2016	225	132	95		
D15A2017	204	201	107		
D15A2018	61	33	18		
D15A2019	152	130	54		
D15A2020	156	144	125		
D15A2021	218	164	115		
D15A2022	223	166	50		
D15A2023	185	161	120		
D15A2024	188	157	134		
D15A2025	171	150	72		
D15A2026	219	159	112		
D15A2027	222	183	112		
D15A2028	209	183	74		
D15A2029	95	75	42		
D15A2030	160	125	55		
D15A2031	191	140	139		
D15A2032	219	152	88		
D15A2033	232	167	123		
D15A2034	157	131	42		
D15A2035	137	128	81		
D15A2036	155	125	101		
D15A2037	180	239	178		
D15A2038	127	197	47		
D15A2039	177	165	122		
D15A2040	174	195	191		
D15A2041	168	157	63		
D15A2042	202	247	132		
D15A2043	178	186	149		
D15A2044	218	234	166		
D15A2045	229	199	95		
D15A2046	188	213	144		
D15A2047	222	212	110		
D15A2048	183	217	109		
D15A2049	225	251	128		
D15A2050	137	248	53		
D15A2051	219	192	85		

D15A2052	213	219	172		
D15A2053	257	245	174		
D15A2054	211	219	147		
D15A2055	231	225	189		
D15A2056	223	214	144		
D15A2057	216	223	111		
D15A2058	241	240	137		
D15A2059	240	231	105		
D15A2060	209	121	66		
D15A2061	227	228	159		
D15A2062	237	220	176		
D15A2063	171	235	100		
D15A2064	176	157	163		
D15A2065	210	180	138		
D15A2066	222	236	58		
D15A2067	211	235	187		
D15A2068	195	255	173		
D15A2069	231	208	92		
D15A2070	206	242	101		
D15A2071	185	236	112		
D15A2072	211	165	139		
D15A2073	210	230	142		
D15A2074	240	248	194		
D15A2075	264	199	92		
D15A2076	245	243	165		
D15A2077	200	161	35		
D15A2078	115	107	34		
D15A2079	185	147	111		
D15A2080	210	214	93		
D15A2081	230	234	192		
D15A2082	204	225	49		
D15A2083	238	241	189		
D15A2084	143	187	125		
D15A2085	190	203	123		
D15A2086	203	211	58		
D15A2087	157	226	126		
D15A2088	103	52	34		
D15A2089	115	120	70		
D15A2090	122	111	22		
D15A2091	94	85	32		
D15A2092	62	49	11		
D15A2093	42	100	71		
D15A2094	167	172	137		
D15A2095	144	156	105		
D15A2096	153	143	93		
D15A2097	128	108	85		
D15A2098	183	121	86		
D15A2099	126	96	118		
D15A2100	176	128	99		
D15A2101	184	124	85		
D15A2102	135	156	76		
D15A2103	189	175	98		
D15A2104	166	203	150		
D15A2105	175	127	37		
D15A2106	190	90	49		
D15A2107	138	105	57		
D15A2108	163	133	121		
D15A2109	125	214	146		
D15A2110	69	140	59		
D15A2111	169	168	135		

D15A2112	152	146	51		
D15A2113	168	171	73		
D15A2114	185	214	66		
D15A2115	113	162	114		
D15A2116	164	168	45		
D15A2117	151	154	100		
D15A2118	141	111	141		
D15A2119	181	124	69		
D15A2120	199	133	92		
D15A2121	150	172	108		
D15A2122	173	135	92		
D15A2123	173	168	81		
D15A2124	161	156	94		
D15A2125	124	154	140		
D15A2126	182	188	119		
D15A2127	181	146	64		
D15A2128	165	177	98		
D15A2129	160	133	112		
D15A2130	138	168	40		
D15A2131	187	155	65		
D15A2132	196	127	85		
D15A2133	177	164	119		
D15A2134	200	124	95		
D15A2135	107	115	83		
D15A2136	113	122	108		
D15A2137	172	107	91		
D15A2138	109	97	60		
D15A2139	174	196	114		
D15A2140	154	148	74		
D15A2141	179	169	94		
D15A2142	166	132	134		
D15A2143	129	175	82		
D15A2144	199	139	36		
D15A2145	185	154	95		
D15A2146	181	124	84		
D15A2147	147	179	47		
D15A2148	175	220	128		
D15A2149	164	175	94		
D15A2150	174	125	125		
D15A2151	204	158	101		
D15A2152	217	143	108		
D15A2153	180	207	87		
D15A2154	105	106	27		
D15A2155	122	168	34		
D15A2156	110	145	85		
D15A2157	72	104	98		
D15A2158	209	243	139		
D15A2159	175	172	82		
D15A2160	183	161	100		
D15A2161	198	208	95		
D15A2162	160	130	66		
D15A2163	220	134	77		
D15A2164	162	176	130		
D15A2165	168	217	93		
D15A2166	172	193	85		
D15A2167	72	216	75		
D15A2168	134	200	129		
D15A2169	151	171	90		
D15A2170	223	139	131		
D15A2171	168	172	29		

D15A2172	179	193	158		
D15A2173	155	206	38		
D15A2174	185	167	126		
D15A2175	171	191	124		
D15A2176	146	223	176		
D15A2177	202	231	163		
D15A2178	153	199	123		
D15A2179	204	153	123		
D15A2180	221	224	158		
D15A2181	233	180	147		
D15A2182	152	150	95		
D15A2183	165	134	125		
D15A2184	221	180	109		
D15A2185	169	195	150		
D15A2186	222	160	113		
D15A2187	209	220	110		
D15A2188	230	181	56		
D15A2189	180	130	38		
D15A2190	203	162	86		
D15A2191	198	133	48		
D15A2192	191	125	78		
D15A2193	214	202	185		
D15A2194	217	163	33		
D15A2195	223	149	88		
D15A2196	200	136	43		
D15A2197	143	121	89		
D15A2198	201	184	142		
D15A2199	142	163	30		
D15A2200	207	181	105		
D15A2201	169	151	125		
D15A2202	175	158	106		
D15A2203	113	190	105		
D15A2204	164	111	74		
D15A2205	195	138	105		
D15A2206	170	186	118		
D15A2207	192	202	122		

H-scores of AGO2, E-cadherin and vimentin in cancer tissues					
Patient ID	AGO2	E-Cadherin	Vimentin		
RDgCol0804A0990	85	87	41		
RDgCol0804A0991	145	81	42		
RDgCol0804A0992	127	89	48		
RDgCol0804A0993	94	89	66		
RDgCol0804A0994	142	106	56		
RDgCol0804A0995	178	137	3		
RDgCol0804A0996	192	66	59		
RDgCol0804A0997	163	97	11		
RDgCol0804A0998	155	84	39		
RDgCol0804A0999	176	84	26		
RDgCol0804A1000	181	171	38		
RDgCol0804A1001	168	59	11		
RDgCol0804A1002	206	171	13		
RDgCol0804A1003	187	128	12		
RDgCol0804A1004	160	158	16		
RDgCol0804A1005	141	207	24		
RDgCol0804A1006	131	103	51		
RDgCol0804A1007	172	158	11		
RDgCol0804A1008	187	201	54		
RDgCol0804A1009	211	136	64		

RDgCol0804A1010	230	125	45		
RDgCol0804A1011	229	103	55		
RDgCol0804A1012	180	136	52		
RDgCol0804A1013	75	100	46		
RDgCol0804A1014	187	31	51		
RDgCol0804A1015	205	150	9		
RDgCol0804A1016	222	214	25		
RDgCol0804A1017	198	115	46		
RDgCol0804A1018	217	192	53		
RDgCol0804A1019	206	171	28		
RDgCol0804A1020	184	140	11		
RDgCol0804A1021	128	227	49		
RDgCol0804A1022	154	105	19		
RDgCol0804A1023	210	181	10		
RDgCol0804A1024	234	227	20		
RDgCol0804A1025	200	91	60		
RDgCol0804A1026	200	193	77		
RDgCol0804A1027	212	120	67		
RDgCol0804A1028	55	74	60		
RDgCol0804A1029	166	99	4		
RDgCol0804A1030	225	85	67		
RDgCol0804A1031	202	116	19		
RDgCol0804A1032	201	9	58		
RDgCol0804A1033	231	92	87		
RDgCol0804A1034	204	119	13		
RDgCol0804A1035	33	26	62		
RDgCol0804A1036	152	223	76		
RDgCol0804A1037	144	221	60		
RDgCol0804A1038	219	42	27		
RDgCol0804A1039	155	220	5		
RDgCol0804A1040	164	178	21		
RDgCol0804A1041	166	200	69		
RDgCol0804A1042	94	183	57		
RDgCol0804A1043	168	169	9		
RDgCol0804A1044	177	161	19		
RDgCol0804A1045	185	79	0		
RDgCol0804A1046	188	192	11		
RDgCol0804A1047	150	105	82		
RDgCol0804A1048	219	111	12		
RDgCol0804A1049	222	140	29		
RDgCol0804A1050	209	69	85		
RDgCol0804A1051	75	187	76		
RDgCol0804A1052	125	216	55		
RDgCol0804A1053	191	50	79		
RDgCol0804A1054	219	87	10		
RDgCol0804A1055	167	174	19		
RDgCol0804A1056	191	102	13		
RDgCol0804A1057	184	105	19		
RDgCol0804A1058	146	83	49		
RDgCol0804A1059	131	174	67		
RDgCol0804A1060	128	185	61		
RDgCol0804A1061	156	179	53		
RDgCol0804A1062	207	167	21		
RDgCol0804A1063	155	203	43		
RDgCol0804A1064	239	193	22		
RDgCol0804A1065	213	183	31		
RDgCol0804A1066	197	195	24		
RDgCol0804A1067	165	245	60		
RDgCol0804A1068	195	252	61		
RDgCol0804A1069	157	160	22		

RDgCol0804A1070	247	24	42		
RDgCol0804A1071	186	211	15		
RDgCol0804A1072	147	194	64		
RDgCol0804A1073	234	211	85		
RDgCol0804A1074	199	201	74		
RDgCol0804A1075	246	110	14		
RDgCol0804A1076	213	250	27		
RDgCol0804A1077	200	88	11		
RDgCol0804A1078	233	155	17		
RDgCol0804A1079	212	178	34		
RDgCol0804A1080	217	163	34		
RDgCol0804A1081	251	171	64		
RDgCol0804A1082	249	223	31		
RDgCol0804A1083	218	172	41		
RDgCol0804A1084	248	171	46		
RDgCol0804A1085	192	170	31		
RDgCol0804A1086	219	208	16		
RDgCol0804A1087	245	194	29		
RDgCol0804A1088	219	240	19		
RDgCol0804A1089	225	185	16		
RDgCol0804A1090	219	163	73		
RDgCol0804A1091	248	171	19		
RDgCol0804A1092	111	99	18		
RDgCol0804A1093	214	161	22		
RDgCol0804A1094	223	164	35		
RDgCol0804A1095	240	242	37		
RDgCol0804A1096	231	212	26		
RDgCol0804A1097	126	216	41		
RDgCol0804A1098	121	176	18		
RDgCol0804A1099	228	113	21		
RDgCol0804A1100	220	205	92		
RDgCol0804A1101	235	152	31		
RDgCol0804A1102	157	195	17		
RDgCol0804A1103	180	146	23		
RDgCol0804A1104	236	171	83		
RDgCol0804A1105	235	103	60		
RDgCol0804A1106	255	119	25		
RDgCol0804A1107	208	97	45		
RDgCol0804A1108	242	246	34		
RDgCol0804A1109	236	171	44		
RDgCol0804A1110	165	239	75		
RDgCol0804A1111	230	116	22		
RDgCol0804A1112	248	86	40		
RDgCol0804A1113	199	163	57		
RDgCol0804A1114	243	142	36		
RDgCol0804A1115	161	157	32		
RDgCol0804A1116	107	202	57		
RDgCol0804A1117	147	206	54		
RDgCol0804A1118	214	120	27		
RDgCol0804A1119	234	234	62		
RDgCol0804A1120	225	169	12		
RDgCol0804A1121	241	116	28		
RDgCol0804A1122	92	142	56		
RDgCol0804A1123	187	139	64		
RDgCol0804A1124	203	188	33		
RDgCol0804A1125	211	100	80		
RDgCol0804A1126	226	197	44		
RDgCol0804A1127	247	95	82		
RDgCol0804A1128	233	200	59		
RDgCol0804A1129	52	142	48		

RDgCol0804A1130	120	107	60		
RDgCol0804A1131	111	99	58		
RDgCol0804A1132	85	143	64		
RDgCol0804A1133	49	137	46		
RDgCol0804A1134	100	70	44		
RDgCol0804A1135	118	112	43		
RDgCol0804A1136	75	50	56		
RDgCol0804A1137	37	122	61		
RDgCol0804A1138	172	134	6		
RDgCol0804A1139	127	85	97		
RDgCol0804A1140	156	136	1		
RDgCol0804A1141	143	78	93		
RDgCol0804A1142	108	85	33		
RDgCol0804A1143	121	199	58		
RDgCol0804A1144	123	191	32		
RDgCol0804A1145	96	152	51		
RDgCol0804A1146	128	81	54		
RDgCol0804A1147	124	113	49		
RDgCol0804A1148	156	104	81		
RDgCol0804A1149	175	162	17		
RDgCol0804A1150	203	129	13		
RDgCol0804A1151	152	115	43		
RDgCol0804A1152	119	204	79		
RDgCol0804A1153	127	125	96		
RDgCol0804A1154	90	120	94		
RDgCol0804A1155	105	120	47		
RDgCol0804A1156	133	63	66		
RDgCol0804A1157	214	145	79		
RDgCol0804A1158	140	132	36		
RDgCol0804A1159	168	89	63		
RDgCol0804A1160	146	138	75		
RDgCol0804A1161	150	140	55		
RDgCol0804A1162	171	132	6		
RDgCol0804A1163	214	114	1		
RDgCol0804A1164	162	105	57		
RDgCol0804A1165	168	104	11		
RDgCol0804A1166	154	79	53		
RDgCol0804A1167	130	48	73		
RDgCol0804A1168	111	168	2		
RDgCol0804A1169	124	116	75		
RDgCol0804A1170	176	148	69		
RDgCol0804A1171	133	45	82		
RDgCol0804A1172	172	140	23		
RDgCol0804A1173	136	176	13		
RDgCol0804A1174	135	107	81		
RDgCol0804A1175	168	200	30		
RDgCol0804A1176	156	61	35		
RDgCol0804A1177	154	174	10		
RDgCol0804A1178	188	155	11		
RDgCol0804A1179	146	126	42		
RDgCol0804A1180	97	84	46		
RDgCol0804A1181	177	114	34		
RDgCol0804A1182	133	88	38		
RDgCol0804A1183	188	179	43		
RDgCol0804A1184	113	124	12		
RDgCol0804A1185	194	136	2		
RDgCol0804A1186	168	93	13		
RDgCol0804A1187	155	36	52		
RDgCol0804A1188	118	46	70		
RDgCol0804A1189	127	93	69		

RDgCol0804A1190	132	34	62		
RDgCol0804A1191	186	134	11		
RDgCol0804A1192	164	63	18		
RDgCol0804A1193	124	160	5		
RDgCol0804A1194	115	135	93		
RDgCol0804A1195	162	104	33		
RDgCol0804A1196	116	126	39		
RDgCol0804A1197	122	101	37		
RDgCol0804A1198	107	101	55		
RDgCol0804A1199	97	148	40		
RDgCol0804A1200	196	68	52		
RDgCol0804A1201	148	170	12		
RDgCol0804A1202	169	132	21		
RDgCol0804A1203	132	121	10		
RDgCol0804A1204	175	103	61		
RDgCol0804A1205	149	125	51		
RDgCol0804A1206	139	89	41		
RDgCol0804A1207	154	110	71		
RDgCol0804A1208	124	135	69		
RDgCol0804A1209	179	93	22		
RDgCol0804A1210	220	144	54		
RDgCol0804A1211	175	35	28		
RDgCol0804A1212	125	128	49		
RDgCol0804A1213	158	81	88		
RDgCol0804A1214	143	166	17		
RDgCol0804A1215	207	76	32		
RDgCol0804A1216	106	218	79		
RDgCol0804A1217	168	211	68		
RDgCol0804A1218	145	71	95		
RDgCol0804A1219	104	147	2		
RDgCol0804A1220	170	153	35		
RDgCol0804A1221	243	181	22		
RDgCol0804A1222	172	143	68		
RDgCol0804A1223	161	88	59		
RDgCol0804A1224	208	162	71		
RDgCol0804A1225	130	130	54		
RDgCol0804A1226	134	165	51		
RDgCol0804A1227	176	128	78		
RDgCol0804A1228	217	171	70		
RDgCol0804A1229	193	143	80		
RDgCol0804A1230	216	206	26		
RDgCol0804A1231	200	114	34		
RDgCol0804A1232	172	62	21		
RDgCol0804A1233	171	169	22		
RDgCol0804A1234	139	73	49		
RDgCol0804A1235	172	164	18		
RDgCol0804A1236	193	159	29		
RDgCol0804A1237	206	146	43		
RDgCol0804A1238	167	119	19		
RDgCol0804A1239	191	209	22		
RDgCol0804A1240	223	94	66		
RDgCol0804A1241	231	77	17		
RDgCol0804A1242	199	73	28		
RDgCol0804A1243	153	190	15		
RDgCol0804A1244	224	142	69		
RDgCol0804A1245	180	199	22		
RDgCol0804A1246	150	239	23		
RDgCol0804A1247	134	132	8		
RDgCol0804A1248	180	96	20		
RDgCol0804A1249	195	165	29		

RDgCol0804A1250	160	212	28		
RDgCol0804A1251	220	143	63		
RDgCol0804A1252	181	234	23		
RDgCol0804A1253	130	152	44		
RDgCol0804A1254	162	182	22		
RDgCol0804A1255	133	91	93		
RDgCol0804A1256	125	130	35		
RDgCol0804A1257	202	117	63		
RDgCol0804A1258	163	143	47		
RDgCol0804A1259	149	169	40		
RDgCol0804A1260	136	108	47		
RDgCol0804A1261	121	190	34		
RDgCol0804A1262	184	79	45		
RDgCol0804A1263	163	228	27		
RDgCol0804A1264	181	161	28		
RDgCol0804A1265	151	81	10		
RDgCol0804A1266	158	100	56		
RDgCol0804A1267	190	103	62		
RDgCol0804A1268	111	101	2		
RDgCol0804A1269	182	136	42		
RDgCol0804A1270	152	111	58		
RDgCol0804A1271	138	195	44		
RDgCol0804A1272	186	142	11		
RDgCol0804A1273	202	113	36		

SW1116-shAGO2/shCON RNA-seq result					
Gene ID	ShCON FPKM	ShAGO2 FPKM	og2(ShAGO2/Shcon	Qvalue	Pvalue
EIF3CL	0	0.843	8.113484172	2.71E-29	2.19E-30
RTL1	0.003	0.213	5.562059786	4.62E-12	9.48E-13
CT45A1	0	0.44	5.522224433	1.78E-06	6.47E-07
KLK6	0	0.263	5.329399029	8.61E-06	3.40E-06
FSBP	0	0.056	5.050990428	6.08E-05	2.73E-05
PPIAL4A	0	0.35	4.810697971	0.00026	0.00013
ADGRL4	0.006	0.143	4.762358436	3.14E-07	1.03E-07
SPP1	0	0.146	4.592433435	0.00079	0.00043
COL6A3	0.003	0.053	4.592433435	1.74E-06	6.29E-07
PCDHGC3	0	0.08	4.549728984	0.00097	0.00053
XAGE1E	0.063	0.853	3.744436528	4.01E-05	1.75E-05
XAGE1B	0.063	0.853	3.744436528	4.01E-05	1.75E-05
CARD11	0.006	0.1	3.53103289	1.60E-05	6.57E-06
KRT19	0.17	1.66	3.2839676	1.29E-23	1.32E-24
FLNC	0.043	0.403	3.263047199	4.55E-35	2.95E-36
VIP	0.026	0.206	3.094933775	0.00048	0.00025
CES1	0.04	0.323	3.094933775	6.48E-07	2.22E-07
ADAM19	0.006	0.046	3.007470934	0.00084	0.00046
MAGEA2	0.056	0.453	2.798964327	1.55E-07	4.89E-08
MYH15	0.06	0.41	2.795966829	2.00E-24	1.99E-25
CEACAM1	0.02	0.12	2.71581585	0.00037	0.00019
TPSG1	0.11	0.693	2.710231964	3.73E-07	1.24E-07
COL13A1	0.033	0.216	2.685542839	7.30E-06	2.85E-06
FBP1	0.04	0.24	2.670435947	0.00061	0.00032
CDRT4	0.136	0.856	2.638178354	4.25E-17	6.12E-18
TEX29	0.12	0.706	2.592433435	2.09E-05	8.72E-06
RAC2	0.066	0.38	2.592433435	2.09E-05	8.72E-06
KIF1A	0.006	0.046	2.481326189	0.00052	0.00027
KIAA1462	0.033	0.173	2.469852673	2.83E-12	5.70E-13
SERPINA1	0.05	0.263	2.466902553	4.84E-07	1.63E-07
DNAH10	0.006	0.036	2.440430342	9.84E-05	4.56E-05
PAEP	0.223	1.17	2.422508434	1.93E-07	6.19E-08

KCNQ3	0.023	0.12	2.383651014	7.01E-10	1.74E-10
HMGA2	3.506	15.97	2.360563101	0	0
DMBT1	0.016	0.083	2.329399029	0.00027	0.00013
U2AF1L5	1.283	6.163	2.299233878	1.09E-39	6.10E-41
ABCG2	0.083	0.393	2.283105377	2.31E-12	4.64E-13
IL24	0.096	0.43	2.280717091	2.59E-06	9.57E-07
CGB8	0.616	2.976	2.250390496	1.37E-17	1.91E-18
VNN1	0.05	0.233	2.240131691	9.15E-07	3.18E-07
VCAN	0.016	0.073	2.209104795	0.00073	0.00039
ADGRF4	0.14	0.626	2.184348696	3.74E-13	7.03E-14
SERPINE2	29.486	127.52	2.113968993	0	0
CGB7	0.146	0.596	2.031027898	0.00057	0.0003
NT5E	1.796	7.093	1.978056205	#######	#######
DRGX	0.183	0.703	1.925008774	0.00017	7.94E-05
CNTN1	0.156	0.57	1.873169843	1.18E-16	1.75E-17
GRPR	0.186	0.673	1.861620068	5.70E-10	1.40E-10
TF	0.066	0.243	1.855467841	0.00016	7.45E-05
MAGEB17	0.816	2.9	1.839916837	6.97E-20	8.52E-21
TFPI	0.143	0.623	1.730689014	3.70E-08	1.09E-08
ALDH1A1	0.076	0.266	1.729936959	0.00057	0.0003
CPA4	14.246	44.62	1.641651154	0	0
ITGAD	0.096	0.3	1.63973915	4.83E-06	1.85E-06
ALOX5AP	0.3	0.856	1.522044107	0.00054	0.00028
CD163L1	0.63	1.796	1.521572561	5.39E-32	3.90E-33
MMP2	0.09	0.256	1.492897761	0.00047	0.00025
GXYLT2	0.376	1.04	1.47561977	9.30E-07	3.24E-07
KIAA0408	0.036	0.096	1.466051757	0.00053	0.00028
ALB	0.136	0.376	1.443570049	0.00036	0.00018
MYEOV	1.103	2.886	1.440028533	1.76E-24	1.75E-25
TGM4	0.116	0.313	1.403111043	0.00027	0.00013
IL7R	1.92	5.09	1.399350713	2.14E-78	4.83E-80
MRAP2	0.2	0.526	1.390799574	7.18E-05	3.26E-05
C5AR2	0.666	1.723	1.362800078	1.61E-08	4.56E-09
ISM1	0.253	0.65	1.353921348	1.96E-06	7.14E-07
GPRC5B	1.56	3.9	1.323400289	1.78E-35	1.14E-36
TRAF1	0.353	0.893	1.321981558	3.28E-12	6.65E-13
LVRN	1.25	3.083	1.308365329	2.78E-43	1.39E-44
ACTL8	1.273	3.143	1.301654039	2.39E-18	3.20E-19
INPP4B	0.28	0.75	1.297170839	1.12E-17	1.55E-18
GAGE2D	12.606	30.776	1.296151308	2.83E-48	1.25E-49
ATP1A3	0.286	0.7	1.274951245	3.63E-08	1.07E-08
IL31RA	0.663	1.59	1.257843298	1.42E-13	2.58E-14
PADI1	0.38	0.91	1.255989999	8.68E-11	1.99E-11
RASD2	0.353	0.83	1.244510132	5.16E-08	1.55E-08
DPYSL3	0.09	0.2	1.229863356	0.0005	0.00026
SOX9	1.446	3.366	1.22366709	1.92E-36	1.18E-37
COL17A1	0.093	0.22	1.222483825	0.00019	9.09E-05
COL6A2	1.726	3.993	1.216417825	7.28E-37	4.39E-38
CREB3L3	0.31	0.72	1.215066354	5.10E-06	1.96E-06
OASL	1.193	2.78	1.185290019	2.27E-15	3.66E-16
SULT1A3	6.86	15.35	1.176264087	1.40E-54	5.37E-56
SOX17	0.366	0.823	1.173829321	5.99E-06	2.31E-06
AMTN	0.48	1.08	1.170969667	0.00082	0.00045
DOCK2	0.653	1.453	1.153650485	1.18E-22	1.27E-23
ANKRD1	2.86	6.333	1.148051974	3.40E-31	2.53E-32
MYOM3	0.116	0.26	1.134351461	0.00011	4.94E-05
MEIOB	0.573	1.26	1.12625619	1.73E-06	6.27E-07
PCLO	0.166	0.363	1.112026865	2.01E-15	3.24E-16
TRIML2	4.98	10.71	1.110857723	1.06E-40	5.75E-42
COL8A1	0.576	1.243	1.105960798	1.25E-09	3.19E-10

ANTXR2	0.436	0.996	1.104153218	8.10E-16	1.27E-16
AMIGO2	4.53	9.643	1.102030633	3.80E-70	1.01E-71
AFP	0.35	0.76	1.099393424	0.00013	5.98E-05
ROBO3	0.266	0.573	1.094933775	6.37E-07	2.18E-07
IL11	2.586	5.386	1.08007074	1.54E-28	1.29E-29
MYLK2	0.22	0.463	1.073560125	0.00064	0.00034
FST	7.383	15.453	1.068039811	1.08E-62	3.45E-64
P2RX6	0.36	0.746	1.062966047	1.91E-05	7.92E-06
PKIB	0.766	1.606	1.06296435	6.96E-08	2.12E-08
DUSP4	7.706	16.026	1.059869619	#######	#######
RIMS2	0.383	0.796	1.05416417	2.07E-07	6.66E-08
MFSD2A	0.3	0.576	1.051865054	0.00087	0.00047
OAF	10.713	22.14	1.047892509	5.21E-90	9.21E-92
SLIT2	0.92	1.873	1.020262488	4.34E-31	3.26E-32
NRP1	4.566	8.116	1.01507075	5.52E-87	1.05E-88
CAPN5	0.47	0.943	1.014134665	2.92E-09	7.75E-10
CBWD6	1.193	2.41	1.010101436	4.25E-09	1.14E-09
IFI44	0.886	1.78	1.007470934	4.47E-07	1.50E-07
KHK	2.65	1.32	-1.000405491	2.38E-13	4.39E-14
CA9	4.173	2.076	-1.001193927	2.73E-13	5.07E-14
LRRC75A	2.116	1.046	-1.00186693	1.98E-12	3.92E-13
TMPRSS3	31.456	17.86	-1.005502277	#######	#######
PTGS2	9.94	4.936	-1.00550692	1.83E-87	3.44E-89
COBL	15.153	7.586	-1.005807061	#######	#######
C1R	18.68	9.286	-1.00780826	2.45E-92	4.08E-94
OC400927-CSNK1	2.16	1.06	-1.011184215	1.34E-13	2.42E-14
SPINT1	0.62	0.3	-1.011388093	0.00051	0.00027
TMEM189-UBE2V1	2.2	1.076	-1.012690149	1.08E-13	1.94E-14
ZXDB	13.026	6.433	-1.020855251	#######	#######
TNNC1	86.58	42.74	-1.021905152	#######	#######
DSC2	0.763	0.376	-1.027462856	4.33E-09	1.16E-09
OTUD7A	0.673	0.526	-1.028152975	7.66E-06	3.00E-06
SLF1	16.44	8.033	-1.035637592	#######	#######
SLC16A14	0.413	0.2	-1.038822718	7.63E-05	3.48E-05
ST6GALNAC2	1.133	0.55	-1.039834781	5.72E-06	2.21E-06
HOTS	18.686	9.073	-1.041909545	#######	#######
NEURL1B	1.163	0.57	-1.041997742	5.69E-15	9.45E-16
PEG10	27.256	13.24	-1.042260925	0	0
PAPPA	1.743	0.846	-1.046210187	7.77E-41	4.18E-42
KLHL13	19.046	9.203	-1.046311736	#######	#######
SAA1	2.61	1.303	-1.048616284	0.00032	0.00016
DCN	21.213	10.283	-1.049448702	#######	#######
LTBP1	18.393	8.93	-1.051912098	#######	#######
PTGDS	2.316	1.116	-1.052650058	4.85E-05	2.13E-05
ARHGAP8	0.83	0.39	-1.05897037	0.00056	0.00029
ZFYVE28	0.66	0.37	-1.059643262	1.94E-06	7.06E-07
SETDB2	2.613	1.25	-1.061423012	2.13E-34	1.43E-35
HOSPHO2-KLHL2	2.183	1.05	-1.064331376	1.71E-20	2.04E-21
FBLN7	17.03	8.01	-1.066034445	6.95E-82	1.48E-83
FCF1	28.283	13.47	-1.066284616	#######	#######
LIPA	24.13	11.486	-1.070437426	#######	#######
WNT6	0.84	0.396	-1.072699414	0.00035	0.00018
RNF182	8.213	3.853	-1.079365003	1.55E-60	5.19E-62
CYP26B1	3.053	1.44	-1.080239304	3.27E-31	2.43E-32
KIAA0040	5.953	2.786	-1.087732319	9.61E-61	3.21E-62
MYO5C	5.063	2.373	-1.091747898	1.82E-79	3.99E-81
MLXIPL	6.723	3.136	-1.096178296	5.59E-50	2.39E-51
LOC102723360	2.863	1.33	-1.097652969	6.13E-15	1.02E-15
TMEM178B	1.83	0.853	-1.099994895	1.58E-44	7.69E-46
SEMA6D	0.413	0.193	-1.104779757	1.14E-06	4.03E-07

WNT1	0.673	0.31	-1.105003795	0.00014	6.47E-05
MMP28	1.073	0.51	-1.105003795	0.00014	6.47E-05
DEPDC1B	14.456	6.69	-1.117508985	2.07E-84	4.14E-86
EPCAM	5.506	2.516	-1.13003259	4.35E-23	4.59E-24
MBNL3	6	2.716	-1.134368386	#######	#######
TJP3	1.543	0.7	-1.136918975	2.03E-12	4.03E-13
GSTA4	1.743	0.79	-1.14190669	1.15E-06	4.07E-07
SYCP2	1.76	0.793	-1.147933649	9.63E-25	9.40E-26
VTN	4.486	2.013	-1.148648268	7.02E-19	9.06E-20
ATP8A1	0.203	0.09	-1.150070343	3.90E-05	1.69E-05
FRAS1	0.153	0.07	-1.159639052	4.46E-07	1.50E-07
ACVR2A	1.26	0.56	-1.160134398	2.69E-17	3.83E-18
NOTUM	12.833	5.716	-1.161505238	7.79E-72	2.01E-73
PLA2R1	0.516	0.24	-1.164844615	3.50E-08	1.03E-08
ABCG1	3.416	1.516	-1.175050712	3.03E-26	2.77E-27
GCOM1	0.816	0.363	-1.18037337	7.73E-12	1.62E-12
CCSER1	0.27	0.12	-1.191646541	0.00021	0.0001
HEY2	0.66	0.286	-1.194162927	1.27E-05	5.15E-06
CACNA1D	0.19	0.08	-1.19606246	7.35E-05	3.35E-05
B4GALNT2	2.036	0.896	-1.197847974	1.10E-10	2.56E-11
HIST3H2BB	2.386	1.03	-1.213273948	0.00089	0.00049
TTC9	2.593	1.113	-1.215597064	1.21E-36	7.40E-38
ENTPD2	2.93	1.253	-1.216392874	1.96E-17	2.76E-18
HOXD8	1.046	0.403	-1.219299928	1.14E-06	4.03E-07
HPD	8.93	3.826	-1.229413774	5.38E-35	3.50E-36
PRODH	4.486	1.95	-1.242666478	2.05E-26	1.86E-27
ASRGL1	11.52	4.856	-1.242791679	4.71E-75	1.14E-76
RNF150	1.733	0.733	-1.243422909	3.98E-23	4.18E-24
NXPH3	0.253	0.103	-1.247786121	5.21E-05	2.31E-05
ELOVL3	2.963	1.243	-1.254270155	8.34E-13	1.61E-13
ERN1	0.773	0.32	-1.27516803	6.75E-10	1.67E-10
PARD6A	3.983	1.63	-1.292514608	1.91E-15	3.08E-16
PARD3B	1.903	0.77	-1.295691584	2.27E-45	1.08E-46
KCNH5	1.57	0.636	-1.296863101	4.27E-19	5.44E-20
C1orf116	0.543	0.22	-1.305119296	1.02E-09	2.56E-10
ADAMTS8	0.366	0.146	-1.305119296	1.87E-05	7.78E-06
ALPPL2	2.936	1.183	-1.307960432	1.75E-22	1.90E-23
CD19	0.486	0.196	-1.313015186	0.00056	0.00029
MPZL2	3.006	1.39	-1.314457161	1.60E-22	1.73E-23
ABCB1	0.206	0.083	-1.325597718	0.00039	0.0002
CHL1	0.463	0.183	-1.32997016	7.40E-12	1.55E-12
SHC2	1.42	0.56	-1.334085967	8.59E-12	1.80E-12
ADAMTSL4	6.466	2.546	-1.337031797	2.40E-83	4.87E-85
PTK7	2.636	1.043	-1.343400116	7.13E-35	4.68E-36
DHRS4L1	0.776	0.303	-1.354389367	0.00014	6.47E-05
GOLGA6L22	0.27	0.1	-1.374959875	0.00023	0.00011
ALPI	29.19	11.24	-1.376610473	#######	#######
TOX3	0.36	0.14	-1.380799655	1.45E-06	5.18E-07
ARHGEF37	0.513	0.2	-1.382095878	4.11E-09	1.10E-09
DTX4	8.156	3.123	-1.393312581	#######	#######
MTPN	12.463	4.706	-1.407577767	#######	#######
COL3A1	1.176	0.443	-1.408985842	2.37E-22	2.58E-23
MUC12	0.433	0.163	-1.413992834	9.77E-25	9.54E-26
ERBB4	0.113	0.04	-1.421372365	1.32E-05	5.36E-06
PRPH	0.523	0.193	-1.435763457	0.00023	0.00011
KLF9	1.536	0.566	-1.436548959	3.74E-28	3.18E-29
GPC5	1.593	0.586	-1.438930736	3.77E-17	5.42E-18
GOLGA8O	0.203	0.073	-1.472372061	6.81E-05	3.08E-05
PXYLP1	2.743	0.936	-1.536849582	4.44E-36	2.77E-37
AGO2	8.963	3.076	-1.543160499	#######	#######

SUMF1	11.443	3.823	-1.577491566	1.82E-97	2.78E-99
PRTN3	0.713	0.236	-1.577491566	0.00078	0.00042
COL25A1	0.136	0.05	-1.577491566	0.00047	0.00024
SPINK5	41.956	14.006	-1.580072747	0	0
SOGA3	0.06	0.023	-1.58387934	0.00049	0.00026
CLDN7	13.42	4.436	-1.585396765	2.56E-83	5.23E-85
GRAMD2	0.436	0.14	-1.655494078	3.45E-07	1.14E-07
WDR64	0.15	0.05	-1.662380464	0.00058	0.00031
YBX2	9.926	3.036	-1.701749669	2.37E-69	6.47E-71
CHRD	0.723	0.22	-1.711792658	2.29E-12	4.60E-13
MISP3	3.753	1.133	-1.725742525	1.13E-22	1.22E-23
MEOX1	1.536	0.48	-1.737487451	1.67E-16	2.51E-17
NDNF	0.286	0.086	-1.751520966	5.99E-05	2.69E-05
PLPPR4	0.156	0.046	-1.76511857	9.94E-05	4.61E-05
SNCA	9.093	2.653	-1.778371767	#######	#######
TNNT2	3.09	0.846	-1.880054337	1.22E-18	1.61E-19
MUC3A	6.686	1.743	-1.944311571	0	0
CP	0.14	0.033	-2.126408255	5.70E-05	2.55E-05
SCN9A	0.91	0.203	-2.148838873	3.75E-54	1.46E-55
FAM47E-STBD1	0.26	0.056	-2.175131457	6.17E-06	2.39E-06
OLFML1	1.856	0.41	-2.189726034	4.04E-34	2.73E-35
INSL4	4.19	0.88	-2.248868819	1.67E-16	2.50E-17
HOXD11	0.53	0.106	-2.314457161	4.22E-06	1.60E-06
MMP7	0.776	0.153	-2.34616602	6.26E-07	2.14E-07
ABCA8	0.133	0.026	-2.380374079	1.66E-06	6.00E-07
CT45A3	0.39	0.066	-2.558326332	0.00058	0.0003
NPNT	40.706	6.746	-2.594429123	0	0
PTPRQ	0.576	0.083	-2.81161328	4.79E-39	2.72E-40
OTOA	0.136	0.013	-3.240456579	0.00017	8.04E-05
DPCR1	0.106	0.01	-3.314457161	1.57E-06	5.63E-07
BHMG1	0.106	0.006	-3.899419661	0.00033	0.00017
FDCSP	0.666	0	-5.240456579	1.58E-05	6.50E-06
HIST1H4J	1.713	0	-5.841026821	7.97E-08	2.44E-08

SW1116-shAGO2/shCON Quantitative proteomics data					
Protein accession	Gene name	Regulated Type	ShAGO2/ShCon Ratio	P value	
P37840	SNCA	Down	0.297	3.92E-05	
P05186	ALPL	Down	0.351	0.011195	
Q9UKV8	AGO2	Down	0.542	7.69E-05	
Q04828	AKR1C1	Down	0.542	0.00896	
P05187	ALPP	Down	0.544	0.001205	
P31327	CPS1	Down	0.552	0.0008	
P25815	S100P	Down	0.555	0.0008	
Q9NWY4	HPF1	Down	0.581	0.000338	
P00966	ASS1	Down	0.581	0.002284	
Q15818	NPTX1	Down	0.592	0.000419	
Q9H479	FN3K	Down	0.604	1.95E-05	
Q96CX2	KCTD12	Down	0.607	4.06E-05	
Q01995	TAGLN	Down	0.61	0.048779	
P29466	CASP1	Down	0.61	0.000959	
O75128	COBL	Down	0.619	0.000982	
Q9Y4D7	PLXND1	Down	0.629	0.002199	
P63316	TNNC1	Down	0.642	0.000557	
O00750	PIK3C2B	Down	0.652	0.012458	
Q3ZCW2	LGALSL	Down	0.652	4.56E-06	
Q9Y6N7	ROBO1	Down	0.653	0.002996	
Q14678	KANK1	Down	0.658	9.83E-05	
Q14134	TRIM29	Down	0.661	0.001059	

Q8IWG1	WDR63	Down	0.671	0.013721	
Q14667	KIAA0100	Down	0.672	0.006795	
Q9H3R2	MUC13	Down	0.673	5.67E-05	
Q9H7S9	ZNF703	Down	0.677	0.000959	
Q9NUK0	MBNL3	Down	0.679	0.002261	
Q14432	PDE3A	Down	0.691	0.000719	
Q86TG7	PEG10	Down	0.693	0.003542	
P23219	PTGS1	Down	0.701	0.018683	
P15941	MUC1	Down	0.701	0.000143	
O75874	IDH1	Down	0.708	0.002738	
P13284	IFI30	Down	0.71	0.000144	
P43007	SLC1A4	Down	0.717	0.009019	
Q9GZY8	MFF	Down	0.718	0.036682	
O75131	CPNE3	Down	0.724	0.000218	
Q96H79	ZC3HAV1L	Down	0.731	9.47E-07	
Q6UUV7	CRTC3	Down	0.734	0.008777	
Q7Z2K6	ERMP1	Down	0.737	4.37E-05	
P27338	MAOB	Down	0.74	0.011258	
Q9UBP0	SPAST	Down	0.742	0.000118	
O96006	ZBED1	Down	0.747	1.9E-05	
Q9ULJ8	PPP1R9A	Down	0.749	0.01008	
Q7L266	ASRGL1	Down	0.751	0.02264	
Q9UJG1	MOSPD1	Down	0.754	0.004177	
Q14244	MAP7	Down	0.755	0.002499	
Q99538	LGMN	Down	0.757	0.003537	
P30047	GCHFR	Down	0.757	0.000882	
Q9Y666	SLC12A7	Down	0.759	0.007738	
O43813	LANCL1	Down	0.761	0.001445	
Q9Y5W7	SNX14	Down	0.761	0.03942	
Q8WX93	PALLD	Down	0.765	0.000882	
Q9H425	C1orf198	Down	0.766	0.013396	
Q8TF42	UBASH3B	Up	1.303	0.013696	
P61225	RAP2B	Up	1.309	7.56E-05	
Q92604	LPGAT1	Up	1.321	5.68E-05	
Q03135	CAV1	Up	1.323	7.89E-05	
Q56VL3	OCIAD2	Up	1.344	0.011358	
P29034	S100A2	Up	1.346	0.018703	
P29323	EPHB2	Up	1.348	0.000362	
Q13424	SNTA1	Up	1.349	0.000663	
P84022	SMAD3	Up	1.354	0.006537	
P58004	SESN2	Up	1.355	0.008757	
O75157	TSC22D2	Up	1.357	0.007065	
Q9UJ68	MSRA	Up	1.365	3.58E-05	
P01137	TGFB1	Up	1.375	0.004743	
O15231	ZNF185	Up	1.375	0.000502	
Q9BZF9	UACA	Up	1.38	6.08E-05	
Q6UWN8	SPINK6	Up	1.38	0.013864	
O14727	APAF1	Up	1.381	0.028299	
Q8NFJ5	GPRC5A	Up	1.381	6.13E-05	
Q9NP84	TNFRSF12A	Up	1.383	0.006138	
Q96B97	SH3KBP1	Up	1.385	0.002342	
Q86SJ2	AMIGO2	Up	1.39	0.045495	
Q92959	SLCO2A1	Up	1.392	0.006639	
Q9Y6M1	IGF2BP2	Up	1.396	2.15E-05	
Q5HY64	FAM47C	Up	1.408	0.047741	
Q9GZQ8	MAP1LC3B	Up	1.409	0.041196	
Q8N1Q1	CA13	Up	1.412	0.034022	
Q02952	AKAP12	Up	1.414	0.000963	
O00622	CYR61	Up	1.442	0.004257	
P17936	IGFBP3	Up	1.447	0.000543	

P17301	ITGA2	Up	1.449	0.011821	
Q00534	CDK6	Up	1.456	0.005804	
Q9UI42	CPA4	Up	1.468	9.5E-05	
P50151	GNG10	Up	1.483	0.009723	
Q5K651	SAMD9	Up	1.491	7.69E-05	
O94907	DKK1	Up	1.567	0.020497	
Q96JB1	DNAH8	Up	1.595	0.000342	
P02795	MT2A	Up	1.633	0.003276	
Q9UFG5	C19orf25	Up	1.698	0.002385	
P55160	NCKAP1L	Up	1.766	0.00236	
O14786	NRP1	Up	1.813	0.000818	
P15144	ANPEP	Up	1.889	0.00024	
Q53H47	SETMAR	Up	2.18	0.000239	
O94885	SASH1	Up	2.329	0.000176	
P52926	HMGA2	Up	4.721	0.000342	

SW1116-shAGO2/shCON miRNA seq data					
sRNA id	Up/Down	log2(shAGO2/shCON)		P value	Q value
hsa-miR-378b	DOWN	-8.108560317		0	0
hsa-miR-378g	DOWN	-6.416242803		0	0
hsa-miR-378e	DOWN	-5.891386095		0	0
hsa-miR-10522-5p	DOWN	-2.409803106		#######	#######
hsa-miR-675-5p	DOWN	-2.355036751		#######	2.43E-99
hsa-miR-3622b-3p	DOWN	-2.044759906		7.55E-84	4.68E-83
hsa-miR-1299	DOWN	-1.809245733		4.56E-23	1.69E-22
hsa-miR-330-5p	DOWN	-1.794699699		0	0
hsa-miR-877-5p	DOWN	-1.718922581		0	0
hsa-miR-548au-5p	DOWN	-1.673839843		1.93E-20	6.72E-20
hsa-miR-188-5p	DOWN	-1.633001427		7.42E-40	3.40E-39
hsa-miR-3529-3p	DOWN	-1.62336851		0	0
hsa-miR-551b-5p	DOWN	-1.619314814		5.47E-23	2.00E-22
hsa-miR-301b-3p	DOWN	-1.616512808		#######	#######
hsa-miR-551b-3p	DOWN	-1.61429618		2.34E-41	1.12E-40
hsa-miR-4479	DOWN	-1.583013291		2.50E-40	1.15E-39
hsa-miR-4466	DOWN	-1.570022862		1.24E-57	6.97E-57
hsa-miR-675-3p	DOWN	-1.566913895		#######	#######
hsa-miR-320e	DOWN	-1.56442583		4.33E-38	1.95E-37
hsa-miR-421	DOWN	-1.555990441		0	0
hsa-miR-766-5p	DOWN	-1.527628388		5.75E-13	1.63E-12
hsa-miR-10526-3p	DOWN	-1.512333068		4.21E-14	1.25E-13
hsa-miR-3177-3p	DOWN	-1.49723136		2.53E-41	1.20E-40
hsa-miR-6765-3p	DOWN	-1.470101613		3.80E-11	9.79E-11
hsa-miR-3186-5p	DOWN	-1.453825788		2.34E-12	6.44E-12
hsa-miR-3620-3p	DOWN	-1.447393591		2.99E-31	1.23E-30
hsa-miR-320b	DOWN	-1.446652545		0	0
hsa-miR-3911	DOWN	-1.434610319		1.75E-25	6.68E-25
hsa-miR-744-5p	DOWN	-1.422034889		0	0
hsa-miR-4286	DOWN	-1.420733223		7.73E-14	2.26E-13
hsa-miR-185-3p	DOWN	-1.416597075		1.44E-18	4.85E-18
hsa-miR-6500-3p	DOWN	-1.414500588		2.46E-18	8.17E-18
hsa-miR-548av-3p	DOWN	-1.405014989		2.40E-14	7.12E-14
hsa-miR-324-5p	DOWN	-1.395198219		0	0
hsa-miR-3158-3p	DOWN	-1.370604958		1.50E-10	3.75E-10
hsa-miR-7706	DOWN	-1.366130851		#######	#######
hsa-miR-301a-3p	DOWN	-1.358807658		0	0
hsa-miR-3187-3p	DOWN	-1.345296524		#######	#######
hsa-miR-330-3p	DOWN	-1.303818928		0	0
hsa-miR-324-3p	DOWN	-1.291279289		0	0
hsa-miR-760	DOWN	-1.288644455		#######	#######

hsa-miR-877-3p	DOWN	-1.259571248		6.59E-23	2.40E-22
hsa-miR-548ad-5p,	DOWN	-1.242420484		1.01E-08	2.31E-08
hsa-miR-3200-3p	DOWN	-1.238998634		3.31E-56	1.81E-55
hsa-miR-320d	DOWN	-1.235241702		0	0
hsa-miR-1266-5p	DOWN	-1.226215049		4.21E-09	9.86E-09
hsa-miR-3679-5p	DOWN	-1.224651119		3.19E-41	1.51E-40
hsa-miR-374c-3p	DOWN	-1.209107461		0	0
hsa-miR-1226-5p	DOWN	-1.208800755		1.48E-18	4.97E-18
hsa-miR-345-5p	DOWN	-1.207088616		0	0
hsa-miR-33a-5p	DOWN	-1.202775128		0	0
hsa-miR-1914-5p	DOWN	-1.182015245		1.16E-07	2.46E-07
hsa-miR-940	DOWN	-1.170789487		#######	#######
hsa-miR-3690	DOWN	-1.170071971		1.18E-15	3.70E-15
hsa-miR-489-3p	DOWN	-1.153466165		1.12E-08	2.53E-08
hsa-miR-10399-3p	DOWN	-1.141155479		7.73E-07	1.51E-06
hsa-miR-423-5p	DOWN	-1.131821135		0	0
hsa-miR-4725-3p	DOWN	-1.126271546		1.57E-19	5.35E-19
hsa-miR-212-5p	DOWN	-1.116210429		2.36E-28	9.31E-28
hsa-miR-185-5p	DOWN	-1.111500031		0	0
hsa-miR-24-2-5p	DOWN	-1.108764013		#######	#######
hsa-miR-4286	DOWN	-1.101285546		#######	#######
hsa-miR-1226-3p	DOWN	-1.098737397		#######	#######
hsa-miR-548b-3p	DOWN	-1.095139144		6.90E-07	1.36E-06
hsa-miR-2110	DOWN	-1.080252144		0	0
hsa-miR-5001-3p	DOWN	-1.076437765		2.18E-07	4.54E-07
hsa-miR-1307-3p	DOWN	-1.068983746		0	0
hsa-miR-4525	DOWN	-1.061060588		2.60E-06	4.86E-06
hsa-miR-7108-5p	DOWN	-1.044647498		6.74E-10	1.64E-09
hsa-miR-3619-5p	DOWN	-1.04348544		2.23E-07	4.62E-07
hsa-miR-548u	DOWN	-1.037661068		1.49E-05	2.66E-05
hsa-miR-570-3p	DOWN	-1.036486371		5.09E-15	1.58E-14
hsa-miR-1260b	DOWN	-1.027587813		8.88E-16	2.80E-15
hsa-miR-1287-5p	DOWN	-1.026197096		#######	#######
hsa-miR-103a-3p	DOWN	-1.023924504		0	0
hsa-miR-3928-3p	DOWN	-1.023585882		2.86E-07	5.89E-07
hsa-miR-3074-5p	DOWN	-1.023423218		0	0
hsa-miR-338-3p	DOWN	-1.022162404		3.16E-80	1.91E-79
hsa-miR-450a-1-3p	DOWN	-1.007644339		2.31E-08	5.14E-08
hsa-miR-326	DOWN	-1.00695152		3.85E-44	1.92E-43
hsa-miR-6747-3p	DOWN	-1.000865806		3.34E-06	6.22E-06
hsa-miR-3622a-5p	UP	1.074658211		7.40E-15	2.27E-14
hsa-miR-139-3p	UP	1.084625622		#######	#######
hsa-miR-487b-3p	UP	1.176637719		2.71E-21	9.69E-21
hsa-miR-323a-3p	UP	1.453000457		8.27E-30	3.36E-29
hsa-miR-409-3p	UP	1.497041976		5.17E-23	1.90E-22
hsa-miR-376a-3p	UP	1.552814605		#######	#######
hsa-miR-379-5p	UP	1.58076196		#######	#######
hsa-miR-543	UP	1.806489116		7.21E-28	2.82E-27
hsa-miR-134-5p	UP	1.810736087		6.75E-19	2.29E-18
hsa-miR-369-5p	UP	1.83794418		1.43E-39	6.54E-39
hsa-miR-376b-3p	UP	1.843209308		#######	#######
hsa-miR-642a-3p	UP	1.8662312		1.26E-31	5.25E-31
hsa-miR-381-3p	UP	1.873899207		#######	2.84E-99
hsa-miR-7-5p	UP	1.887250347		0	0
hsa-miR-369-3p	UP	1.985874447		2.79E-33	1.18E-32
hsa-miR-411-5p	UP	2.036129621		0	0
hsa-miR-411-3p	UP	2.040166539		8.62E-39	3.91E-38
hsa-miR-494-3p	UP	2.042981734		4.57E-42	2.21E-41
hsa-miR-654-3p	UP	2.086038609		8.90E-38	3.95E-37
hsa-miR-376c-3p	UP	2.200381362		0	0

hsa-miR-382-5p	UP	2.232175288		7.29E-35	3.14E-34
hsa-miR-135a-5p	UP	2.478914458		3.58E-43	1.78E-42
hsa-miR-181a-3p	UP	2.667816423		0	0
hsa-miR-378f	UP	6.627608403		0	0
